# Systematic Dissection of Roles for Chromatin Regulators in a Yeast Stress Response

**DOI:** 10.1371/journal.pbio.1001369

**Published:** 2012-07-31

**Authors:** Assaf Weiner, Hsiuyi V. Chen, Chih Long Liu, Ayelet Rahat, Avital Klien, Luis Soares, Mohanram Gudipati, Jenna Pfeffner, Aviv Regev, Stephen Buratowski, Jeffrey A. Pleiss, Nir Friedman, Oliver J. Rando

**Affiliations:** 1 School of Computer Science and Engineering, The Hebrew University, Jerusalem, Israel; 2 Alexander Silberman Institute of Life Sciences, The Hebrew University, Jerusalem, Israel; 3 Department of Biochemistry and Molecular Pharmacology, University of Massachusetts Medical School, Worcester, Massachusetts, United States of America; 4 Department of Biochemistry and Molecular Pharmacology, Harvard Medical School, Boston, Massachusetts, United States of America; 5 Department of Molecular Biology and Genetics, Cornell University, Ithaca, New York, United States of America; 6 Broad Institute of MIT and Harvard, Cambridge, Massachusetts, United States of America; 7 Howard Hughes Medical Institute, Massachusetts Institute of Technology, Cambridge, Massachusetts, United States of America; Stowers Institute, United States of America

## Abstract

Packaging of eukaryotic genomes into chromatin has wide-ranging effects on gene transcription. Curiously, it is commonly observed that deletion of a global chromatin regulator affects expression of only a limited subset of genes bound to or modified by the regulator in question. However, in many single-gene studies it has become clear that chromatin regulators often do not affect steady-state transcription, but instead are required for normal transcriptional reprogramming by environmental cues. We therefore have systematically investigated the effects of 83 histone mutants, and 119 gene deletion mutants, on induction/repression dynamics of 170 transcripts in response to diamide stress in yeast. Importantly, we find that chromatin regulators play far more pronounced roles during gene induction/repression than they do in steady-state expression. Furthermore, by jointly analyzing the substrates (histone mutants) and enzymes (chromatin modifier deletions) we identify specific interactions between histone modifications and their regulators. Combining these functional results with genome-wide mapping of several histone marks in the same time course, we systematically investigated the correspondence between histone modification occurrence and function. We followed up on one pathway, finding that Set1-dependent H3K4 methylation primarily acts as a gene repressor during multiple stresses, specifically at genes involved in ribosome biosynthesis. Set1-dependent repression of ribosomal genes occurs via distinct pathways for ribosomal protein genes and ribosomal biogenesis genes, which can be separated based on genetic requirements for repression and based on chromatin changes during gene repression. Together, our dynamic studies provide a rich resource for investigating chromatin regulation, and identify a significant role for the “activating” mark H3K4me3 in gene repression.

## Abstract 2

Systematic functional and mapping studies of histone modifications in yeast show that most chromatin regulators are more important for dynamic transcriptional reprogramming than for steady-state gene expression.

## Author Summary

Chromatin packaging of eukaryotic genomes has wideranging, yet poorly understood, effects on gene regulation. Curiously, many histone modifications occur on the majority of genes, yet their loss typically affects a small subset of those genes. Here, we examine gene expression defects in 200 chromatin-related mutants during a stress response, finding that chromatin regulators have far greater effects on the dynamics of gene expression than on the steady-state transcription. By grouping mutants according to their shared defects in the stress response, we systematically recover known chromatin-related complexes and pathways, and predict several novel pathways. Finally, by integrating genome-wide changes in the locations of five prominent histone modifications during the stress response with our functional data, we uncover a novel role for the “activating” histone modification H3K4me3 in gene repression. Surprisingly, H3K4 methylation appears to act in conjunction with H3S10 phosphorylation in the repression of ribosomal biosynthesis genes. Repression of ribosomal protein genes and ribosomal RNA maturation genes occur via distinct pathways. Our results show that steady-state studies miss a great deal of important chromatin biology, and identify a surprising role for H3K4 methylation in ribosomal gene repression in yeast.

## Introduction

Packaging of eukaryotic genomes into chromatin has wide-ranging effects on gene transcription in eukaryotes [1]. There are two major ways in which cells modulate nucleosomal influences on gene expression. ATP-dependent chromatin remodeling machines utilize the energy of ATP hydrolysis to disrupt histone-DNA contacts, often resulting in nucleosome eviction and changed nucleosomal location or subunit composition [2]. In addition, the highly conserved histone proteins are subject to multiple types of covalent modification, including acetylation, methylation, phosphorylation, ubiquitination, SUMOylation, and ADP-ribosylation. These covalent histone modifications often occur during the process of transcription, and in turn have many effects on transcription. Moderately well-understood effects of histone modifications include epigenetic gene silencing, control of transcript structure via repression of “cryptic” internal promoters, control of splicing, and transcriptional activation [3]–[7]. Altogether, there are myriad interactions and feedback loops between chromatin state and transcription. At present, the effect of most modifications on transcription is unclear, even for reasonably well-characterized ones.

A large number of systematic genome-wide analyses have been carried out to characterize the complex interplay between chromatin regulation and gene transcription. Genome-wide mapping studies [8],[9] show that modification patterns are correlated with gene structure and gene activity levels. Genome-wide mRNA profiling has been used for over a decade to identify transcriptional defects in chromatin mutants [10]. A recent tour de force from the Holstege lab examined the effects on gene expression of deleting each of 174 different chromatin regulators [11]. Proteomic studies characterize many of the protein complexes that play a role in chromatin regulation [12],[13]. Systematic genetic interaction profiling (using growth rate as a phenotype) has been used to identify chromatin complexes, and to delineate interactions between chromatin pathways [14]–[16]. Importantly, most of these genomic screens have been carried out in steady-state conditions, typically in yeast actively growing in rich media.

In contrast, single gene studies suggest that chromatin regulators have important roles in dynamic processes that are masked at steady-state. For instance, deletions of the histone acetylase Gcn5 or the histone chaperone Asf1 have little effect on the eventual induction of *PHO5* by phosphate starvation, but both of these deletions cause significant delays in *PHO5* induction kinetics [17],[18]. Similarly, mutation of H3K56, whose acetylation plays a role in histone replacement, delays *PHO5* induction by slowing nucleosome eviction upon gene activation [19]. Similar results hold for other classic model genes, such as the galactose-inducible *GAL* genes [20]. Because steady-state gene expression in mutants is subject to widespread compensatory or homeostatic mechanisms, we reasoned that analysis of mutant responses to a stressful stimulus would help reveal direct functions of transcriptional regulators. Thus, the dynamics of response to stimuli should uncover the transcriptional roles of histone-modifying enzymes and other chromatin regulators. We chose diamide stress in yeast as a model system, as it has been shown to involve a rapid, dramatic reorganization of the yeast transcriptome with 602 genes induced more than 2-fold and 593 genes repressed [21].

Here, we carried out a time course of diamide stress in 202 yeast mutants and characterized gene expression changes at 170 selected transcripts (Figure S1A–C). Importantly, analysis of thousands of genome-wide mRNA profiling studies shows that genes typically are co-regulated in coherent clusters [22]–[24], meaning that the behavior of the majority of co-regulated clusters can be captured by analyzing ∼100–200 transcripts. For example, analyzing mutant effects on six ribosomal protein genes suffices to capture the majority of mutant effects on all ∼250 of these genes.

We find that the majority of chromatin regulators have greater effects on gene induction/repression kinetics than they do on steady-state mRNA levels, confirming that dynamic studies can identify unanticipated functions for chromatin regulators. We show that grouping deletion mutants with similar gene expression defects identifies known complexes, and that joint analysis of histone mutants and deletion mutants associates many histone-modifying enzymes with their target sites. In addition to known relationships between chromatin regulators, we identify a number of novel connections, including a previously unknown connection between H3K4 and H3S10 modifications. We further carried out genome-wide mapping of five relevant histone modifications during the same stress time course (Figure S1D–E). By combining functional data with genome-wide mapping data, we identify a key role for Set1-dependent H3K4 methylation in repression of ribosomal biogenesis genes. H3K4 methylation and H3S10 phosphorylation are both required for full repression of ribosomal protein genes (RPG) and of genes involved in rRNA maturation (RiBi), but repression of RPGs and RiBi genes operate via two distinct pathways downstream of these histone marks. Thus, the classic “activating” mark H3K4me3 in fact serves primarily to facilitate repression in budding yeast under multiple stress conditions. Together, these data provide a rich multi-modal view on the role of chromatin regulators in gene induction and repression dynamics, and suggest that understanding the myriad roles of chromatin structure in gene regulation on a genome-wide scale will require extending mutant analyses to kinetic studies.

## Results

### Time Course Analysis of Stress Response in Chromatin Mutants

**Figure 1:**
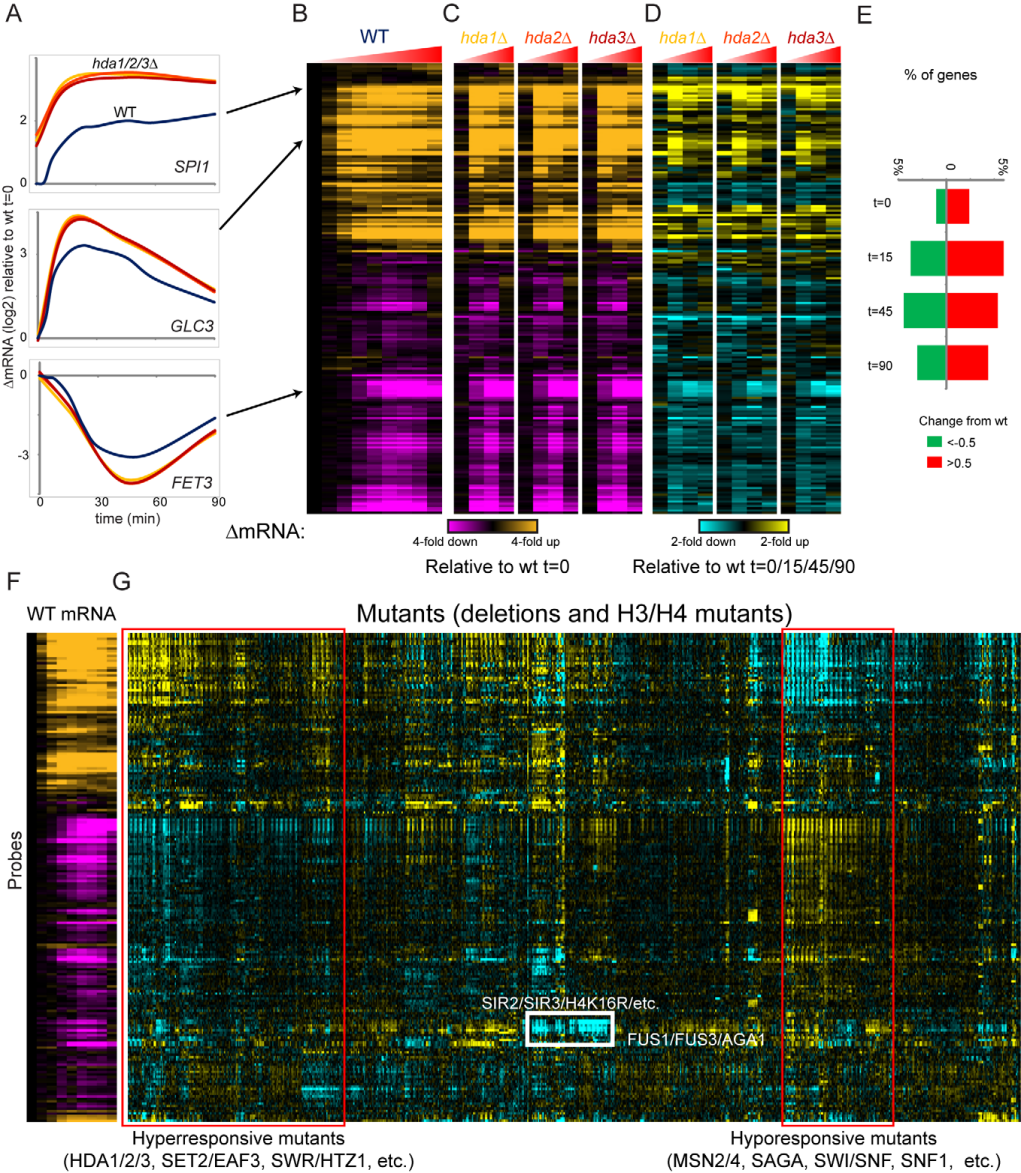
Chromatin mutant effects on mRNA expression dynamics during stress. (A) Time course data for three genes in wild type and three mutant (*hda1*Δ, *hda2*Δ, and *hda3*Δ) yeast. For each time course, data are normalized to wild type t = 0. Wild type time course includes nine time points after diamide addition (0, 4, 8, 15, 22.5, 30, 45, 60, and 90 min after diamide addition), while mutant time courses cover four time points (t = 0, 15, 45, 90). (B) Wild type stress response. Data for all 200 probes are shown as log(2) fold change relative to t = 0, with probes ordered by hierarchical clustering [22]. (C) As in (B), but for the three indicated mutants. As in (B), data are normalized to wild type t = 0. (D) “Difference map” for three mutants. Here, data for the three mutants are normalized relative to the equivalent wild type time point. *hda1*Δ t = 15 is compared to wt t = 15, etc. Note that many more dramatic effects on gene expression are observed during diamide stress than are observed at t = 0. (E) Chromatin mutants have more widespread effects on gene expression during the stress response than during steady-state growth in YPD. Plotted are the fraction of (mutant×probe) effects with increased, or decreased, expression of the probe in question. This number represents the fraction of all entries in the 200 probe×202 mutant matrix (for each time point) with an absolute log2 change in RNA abundance of greater than 0.5. (F–G) Entire dataset for diamide stress. (F) shows wild type data as in (B). (G) shows data for 202 indicated mutants, normalized relative to equivalent wild type time points as in (D). All four time points for each mutant are contiguous, resulting in a “striped” appearance for groups of mutants that specifically affect a subset of time points during diamide stress. Red boxes indicate large groups of mutants that exhibit a widespread decrease (“hyporesponsive”) or increase (“hyperresponsive”) in the amplitude of the overall diamide stress response. White box indicates an example of a subcluster expected from prior knowledge. Mutants in the Sir heterochromatin complex express pheromone response genes at low levels due to the “pseudodiploid” state caused by derepression of the silent mating loci in these mutants. Data are also provided in Table S1.

We used nCounter technology [25] to carry out genome-scale gene expression profiling. Briefly, this technology utilizes hybridization of labeled oligonucleotides in a flow cell to directly count individual RNA molecules, without any enzymatic steps, for several hundred RNAs in yeast extracts. For this experiment, we focused on gene expression during a stress response time course (using the sulfhydryl oxidizing agent diamide). We used whole genome mRNA abundance and Pol2 localization data from prior diamide exposure time courses [21],[26], along with a compendium of prior whole genome mRNA analyses and transcript structure analyses in various mutants [23],[24], to select 200 probes reporting on 170 transcripts (142 genes, of which 30 had two sense probes, as well as another 28 antisense transcription units) that capture the majority of the different patterns of gene expression behavior in this stress. Using this probeset, we measured transcript abundances over a 90-min time course of diamide exposure (Figure 1). Experimental replicates are highly reproducible (Table S1), and these data provide a detailed kinetic perspective on gene expression dynamics during the diamide stress response (Figure 1A–D).

We carried out identical time course experiments for 119 deletion strains for chromatin regulatory genes and for 83 mutants in histones H3 and H4 [27], covering the majority of individual K→R, K→Q, K→A, R→K, and S→A mutants, and several H3 and H4 N-terminal tail deletions. For most mutants, we analyzed mRNA abundance at four time points (*t* = 0, 15, 45, and 90 min) as these time points capture the major phases of the diamide stress response. Figure 1A–D show example data for wild-type yeast and three mutants in the HDA1/2/3 complex. The entire dataset, comprising ∼1,000 experiments carried out for 202 mutant strains, is shown in Figure 1F–G, with mutant time courses clustered according to the similarity between their effects on gene expression across all four time points (see also Table S1).

### Most Chromatin Mutants Have Greater Effects on Gene Induction/Repression Than on Steady-State Expression

Close inspection of the cluster in Figure 1G (Table S1) revealed that many of the gene expression defects observed in these mutants were only observed during the stress response, but not before stress. This is apparent in Figure 1A and 1D, where many more genes exhibit different levels between wild-type and *hda* mutants at 15 and 45 min of the stress response than at t = 0 (midlog growth). These differences include both kinetic delays in gene induction/repression and defects in the extent of gene regulation (see below). To determine the generality of this phenomenon, we determined the distribution of mutant effects on RNA abundance at each of the four time points in the stress response. Many more significant gene expression changes relative to wild-type occur at 15 and 45 min (∼10% of probe/mutant pairwise interactions) after diamide addition than at t = 0 (∼3.5% of pairwise interactions, Figure 1E). As the yeast acclimate to the stress environment (e.g., at t = 90), the transcriptome reaches a new steady-state where we see fewer large mutant effects, although there are still more changes than at t = 0. Thus, consistent with observations from classical model genes such as *PHO5*, we find that chromatin mutants have much more extensive effects during changes in transcription than during steady-state conditions.

### Overall Stress Responsiveness Correlates with Nucleosome Occupancy

We sought to identify major classes of gene expression defect in various chromatin mutants, as a first step in eventually linking chromatin transitions to the genetic requirements for different chromatin regulators. Immediately apparent in Figure 1G (red boxes) are two large groups of mutants with opposing behaviors with respect to the stress response—mutants that appear to be transcriptionally “hyper-responsive” to diamide stress and “hypo-responsive” mutants that exhibit blunted stress responses. These two major classes of mutants are also captured by principal component analysis (PCA) of our dataset. Here, the first principal component, which explains 30% of the variance in the dataset, corresponds to hyper- and hypo-responsive mutants (Figure S2A–B). Interestingly, not all genes induced or repressed during diamide stress were affected by hyper- or hypo-responsive mutants. Genes whose induction was most affected by hyper-responsive mutants, for example, tended to be those with highly nucleosome-occupied promoters in YPD (Figure S2C) [28]–[30].

Hypo-responsive mutants to diamide stress included a number of expected mutants, including deletion mutants lacking the general stress transcription factors Msn2 and Msn4, or with compromised coactivator complexes such as Swi/Snf or SAGA. Hyper-responsive mutants, conversely, included a number of histone deacetylases such as Hda1/2/3. Beyond acetylation/deacetylation, hyper-responsive and hypo-responsive mutants included a variety of deletions known to affect histone turnover and/or occupancy. Several of these factors have previously been shown to affect bulk H3 turnover (Rtt109, Cac2/Rtt106, Htz1, Hat1, Rsc1, and Nhp10; [31]–[37]) or histone levels/occupancy (Rtt109, Yta7, Rtt106, Cac2, Spt21, H3K42Q; [38]–[40]). Interestingly, we noticed that among those histone mutants that decreased the stress response program, the subset of those mutations that are located in the globular domains of H3/H4 (as opposed to the N-terminal tails) are all situated at histone-DNA interfaces (Figure S2D), which we speculate could affect nucleosomal stability and/or replacement dynamics. Taken together, these results support a model in which many chromatin regulators have roles on global transcriptional responsiveness resulting from their overall effects on nucleosome stability.

### Single Cell Analysis of Chromatin Regulation of Gene Expression

Our RNA abundance measurements provide a population-averaged view of chromatin effects on gene expression, but hide a great deal of stochastic behavior that can be revealed by single-cell approaches. For example, RNA data on hyper-responsive mutants come from many thousands of cells, meaning the mechanistic basis for stress hyper-responsiveness is unknown. Do hyper-responsive mutants have a greater fraction of cells exhibiting diamide-driven gene induction (as might be observed if gene induction depends on cell cycle stage and mutants exhibit cell cycle delays), or do all individual cells exhibit greater amplitude responses?

**Figure 2:**
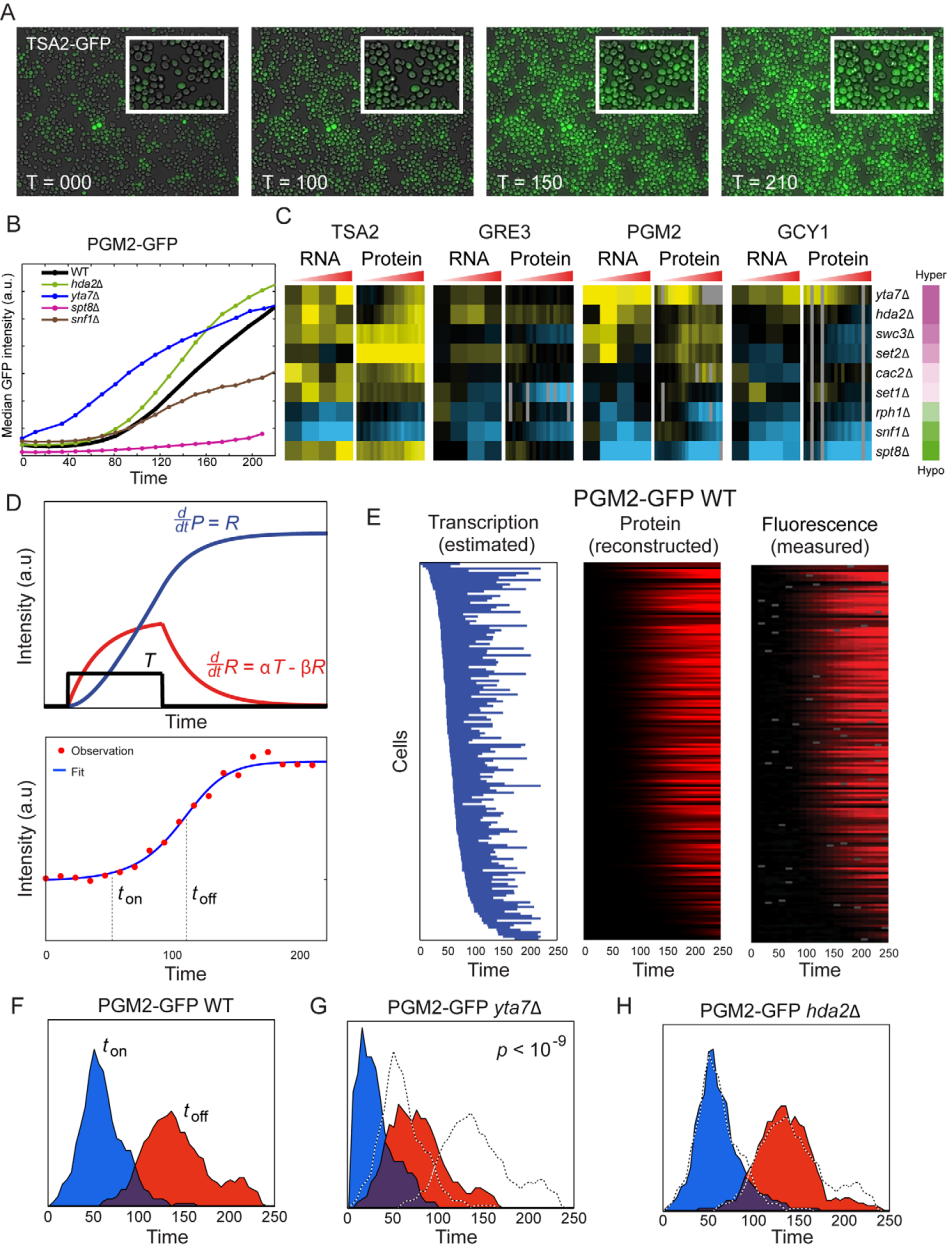
Single cell analysis of mutant effects on gene induction. (A) Sample images from a time-lapse microscope analysis of a Tsa2-GFP fusion reporter at the indicated times after diamide treatment. Midlog yeast cells were grown in a mono-cell layer on glass bottom plate coated with Concavalin-A. The cells are attached to the glass and thus remain at the same location in successive images. Shown is GFP image overlaid on transmitted light image (both at 40× magnification). (B) Time course fluorescence data for Pgm2-GFP for wild-type and the four indicated mutants. Each curve represents median fluorescence versus time for responding cells of a specific strain (*n*∼250±100). (C) Protein expression recapitulates mutant effects on RNA abundance. Data are shown for four GFP fusions, each analyzed in nine deletion mutants. Left panel shows hyper/hypo responsiveness score for the mutant in question (Figure S2). For each of the four promoters, left panel shows RNA data as in Figure 1G, while right panel shows the log-ratio between median GFP expression in wild-type and in a given mutant. (D) Analytical model to extract promoter “open” time and expression rate. A simple model in which cells transition from a low expression state to a high expression state was fit for each cell, resulting in three parameters: *t_on_* (time from diamide treatment to beginning of high expression), *t_off_* (time of return to low expression), and production rate during high expression. Figure shows data (red dots) and fit (blue curve) for a single cell expressing Pgm2-GFP. (E) Model accurately captures GFP expression with few parameters. Left panel shows the duration of the high expression state for Pgm2-GFP (wild-type) as a blue bar, with cells ordered by *t_on_*. Middle panel shows model predictions of protein levels. Right panel shows data for each cell. (F–H) Two hyperresponsive mutants differ in the mechanism for enhanced Pgm2-GFP production. Histograms of single cell distributions for *t_on_* (blue) and *t_off_* (red) are shown for wild-type (F), *yta7*Δ (G), and *hda2*Δ (H). While *yta7*Δ mutants clearly are hyperresponsive due to abnormally rapid gene induction, the minor changes in *t_on_* and *t_off_* in *hda2*Δ mutants suggest that these mutants instead are hyperresponsive to diamide as a result of increased RNA/protein production per unit time during the stress response. This could result from an effect on RNA polymerase burst size or elongation rate, or RNA stability.

We therefore extended our studies to include single cell analysis of protein expression using high throughput microscopy of GFP-tagged proteins in several key mutants. As protein stability significantly confounds measures of gene repression, we focused on four diamide-induced genes, and examined each reporter in wild type and in nine deletion mutants. We conducted time-lapse microscopy of yeast cells during the diamide response (Figure 2A, Methods). After detecting cells (average *n* = 120 for each of 40 strains, two biological replicates), we quantified the temporal profile of GFP intensity for each cell. Figure 2B shows the median intensity as a function of time for one reporter in wild-type and several mutants. Importantly, we found excellent agreement between defects in protein induction in various hypo- and hyper-responsive mutants and the corresponding nCounter RNA measurements (Figure 2C).

In general, we noted that GFP induction in individual cells followed a sigmoid-like curve consistent with a window of stress-increased protein production followed by a gradual return to baseline production levels. This behavior is consistent with a simple model in which there is a time window of diamide-induced gene transcription, followed by gradual mRNA decay. We implemented a simple mathematical model with cells transitioning from low expression to high expression and back, with a constant rate of mRNA production during the open window (Materials and Methods). This model is clearly oversimplified—each parameter covers multiple processes—but provides very good fit to the measured intensity profiles (Figure 2D). Fitting the model for each cell, we can estimate the transcriptional time windows for individual cells as well as the rate of protein production during this time and examine the variability in the timing and speed of transcriptional response in a genetically homogenous population of cells (Figure 2E).

We then used the extracted parameters for individual cells to determine whether hyper- or hypo-responsiveness corresponded to a change in the responsive fraction of cells, a population-wide change in promoter open time, and so forth. In general, we found that most mutants did not affect the fraction of cells responding to diamide. The fraction of cells exhibiting diamide induction of GFP was 87%±3% across all 40 strains, and no strain differed from wild-type by even 10% of cells responding. Notably, we found that different hyper-responsive mutants could act at different stages in gene expression. For example, deletion of *YTA7*, which is involved in histone gene transcription and affects nucleosome occupancy [40],[41], leads to accelerated promoter opening during diamide stress, whereas deletion of *HDA2* predominantly affects GFP production rate rather than promoter opening (Figure 2F,G). Together, these results independently validate our RNA measurements, confirm that RNA changes are reflected in protein abundance, and show that, for the nine mutants analyzed, mutant effects on transcriptional response occur in the majority of cells rather than reflecting changes in the fraction of diamide-responsive cells.

### Similarity Between Mutant Profiles Identifies Complexes and Pathways

**Figure 3:**
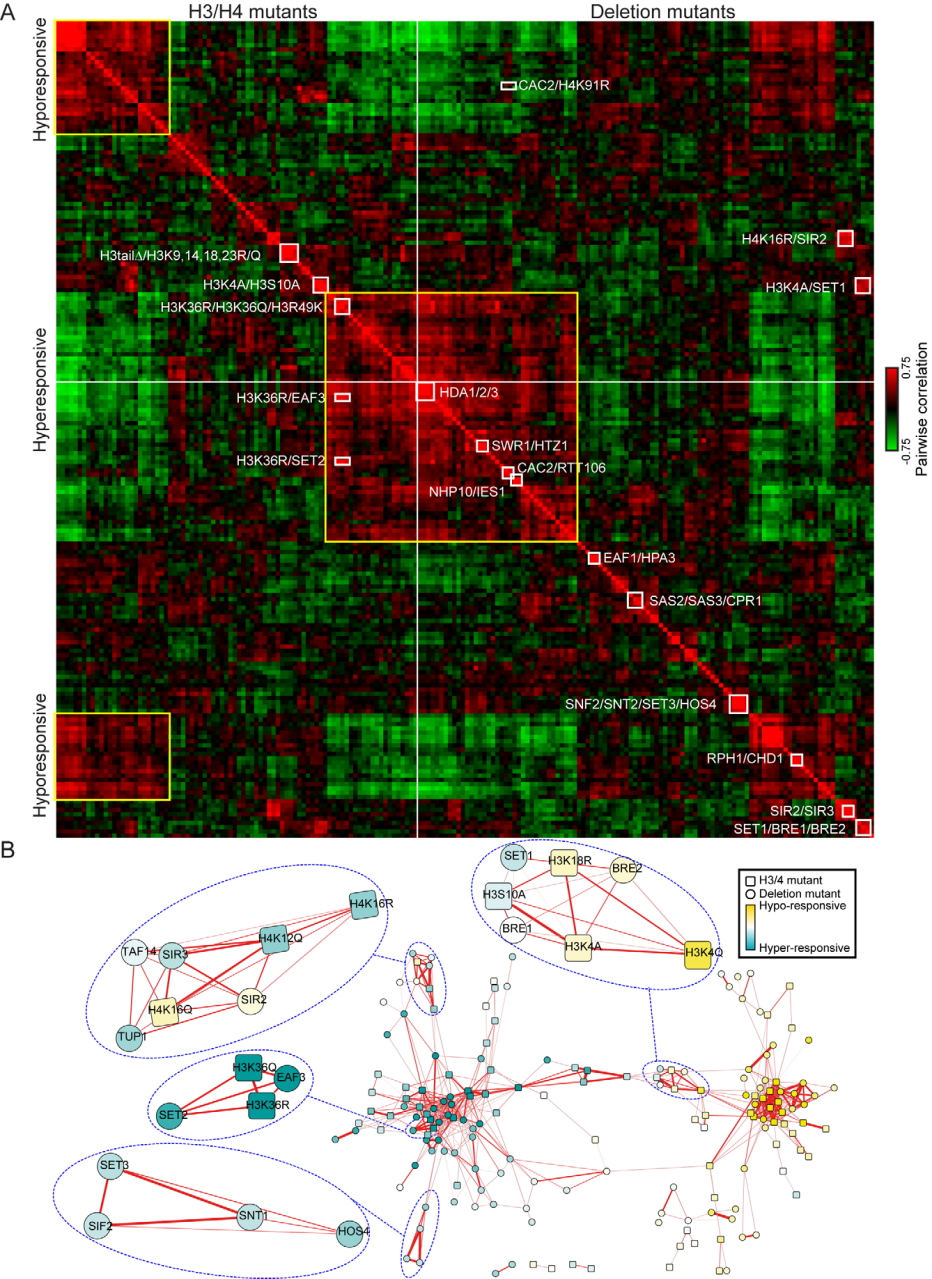
Correlation matrix identifies complex membership and enzyme-substrate relationships. (A) Correlation matrix for all 202 mutants. Correlation between each mutant's effects on diamide stress response was calculated across the entire time course, and mutants were clustered by correlation coefficient. Rows and columns are ordered identically. Note that histone mutants and gene deletion mutants are kept separate, as indicated. Boxes indicate an illustrative subset of highly correlated groups of mutants corresponding to known co-membership in protein complexes (e.g., SIR2/SIR3), known pathways (e.g., SWR1/HTZ1), novel predicted pathways (e.g., RPH1/CHD1), and expected or novel relationships between histone residues and chromatin regulators (e.g., H3K36/SET2/EAF3). (B) Network wiring of chromatin regulators. Genes were grouped according to correlations (thresholded at 0.45), and related genes are clustered using cytoscape implementation of the spring embedded layout [91]. Subnetworks corresponding to several complexes are emphasized as indicated.

Beyond the major groups of mutants that affect overall stress responsiveness and likely report on global histone occupancy/dynamics, we observed a wide variety of gene expression effects that were specific to smaller sets of mutants. For example, the white box in Figure 1G highlights the well-understood gene expression changes that occur in mutants related to the Sir heterochromatin complex—repression of mating-related genes secondary to the pseudodiploid state of these mutants [7]. To systematically group mutants according to their gene expression phenotypes, we calculated the correlations between the changes (relative to wild-type) in stress response in each mutant and clustered mutants according to these correlations (Figure 3A, Table S2, Materials and Methods). We kept histone mutants and deletion mutants separate to allow more intuitive interpretation of clusters.

Grouping deletion mutants by this method recovers a great deal of known chromatin biology, validating our approach. In general, mutants in different subunits of known chromatin complexes exhibit similar defects in gene expression, indicating shared function. Most white boxes in Figure 3A highlight a subset of clear examples, including the grouping of subunits of the Sir complex, the HDA1/2/3 complex, COMPASS, Cac2/Rtt106, Set3C, and the Ino80 complex. Furthermore, several pathways were recovered. The histone variant H2A.Z (encoded by *HTZ1*) was linked to components of the Swr1 complex responsible for H2A.Z incorporation [42]–[44], the H3K4 methylase Set1 was linked to the H2B ubiquitin ligase Bre1 whose activity is required for K4 methylation [45], and the H3K36 methylase Set2 was linked to Eaf3, the binding partner for H3K36me3 [16],[46],[47].

In addition to known chromatin regulatory complexes and pathways, our results also suggest a number of hypotheses for novel chromatin pathways. For example, we find strong correlations between gene expression defects in mutants lacking the H4K16 acetylase Sas2 and those lacking the proline cis/trans isomerase Cpr1. Similarly, our results link the H3K36 demethylase Rph1 with ATP-dependent remodeler Chd1, suggesting the possibility that H3K36 methylation regulates Chd1 in budding yeast, an idea that finds support in prior studies showing that H3K36 mutants and *chd1* mutants have similar genetic interactions in vivo [48].

Analysis of histone mutations revealed similar structure. We observe two larger clusters that correspond to hyper- and hypo-responsive mutations (Figure 3A, yellow boxes), as well as many smaller groups. Many of these groups are comprised of several mutations in the same residue (e.g., all three mutations in H3K36 are tightly clustered together) or in the same tail (e.g., H3 tail delete and simultaneous K->Q/R mutations in H3 tail lysines 4, 9, 14, 18, and 23). Many other groups of histone mutants were unanticipated and may identify functionally relevant nucleosomal surfaces [27] or novel examples of histone crosstalk [49]. Below, we explore the relevance of one such novel connection, between H3K4A and H3S10A mutants.

Many of the connections between chromatin regulatory genes observed here also can be observed in systematic genetic interaction profiles, or in gene expression studies carried out in midlog growth conditions [11],[14]. A unique aspect of our study is the joint analysis of gene deletion mutants with histone point mutants. Many of the strongest correlations between deletion and histone mutants correspond to known enzyme-substrate and modification-binding partner relationships. For example, gene expression defects resulting from deletion of the H3K36 methylase Set2 were most strongly correlated with the defects in H3K36R and H3K36Q mutants, and with the H3K36me3-binding protein Eaf3 (Figure 3A).

Analysis of multiple different mutations of the same lysine residue can provide insight into the biochemical function of modifications at this residue. While both K→R and K→Q mutants disrupt modification-specific binding by proteins (e.g., bromo- and chromo domain proteins), they differ in their charge. Indeed, lysine mutants for which K→R and K→Q mutants exhibited similar gene expression defects tend to occur at lysines with well-characterized modification-specific binding partners (e.g., Eaf3, Sir3). In contrast, lysines for which K→R and K→Q mutants had opposing effects on gene expression often were known acetylation substrates, although we counterintuitively observe that for these lysines the K→R mutations were generally correlated with deletions in histone *de*acetylases (Figure S3).

To systematically identify relationships between chromatin factors, we identified significant correlations between mutants (Materials and Methods), recovering for example the Set2→H3K36→Eaf3 pathway (Figure S4A–B, Table S3). Data for all correlations above a threshold significance are visualized in a network view in Figure 3B to show not only connections within strongly connected pathways but also connections between pathways. Other known relationships recovered this way included the association between Set1 and H3K4, and the association between the Sir complex and H4K16 (Figure S4).

Furthermore, we found that Cac2, a CAF-1 subunit, and Rtt106, histone chaperones that were strongly correlated with one another, exhibited transcriptional effects most related to the H4K91R mutant (Figure 3A). H4K91 acetylation is a little-studied modification reported to occur on newly synthesized histones [50], and in systematic genetic interaction studies, H4K91R and mutations in the assembly-related lysine H3K56 exhibited similar genetic interactions [27]. We therefore hypothesize that H4K91 acetylation might affect chromatin assembly by CAF-1 or Rtt106. Other connections have no obvious literature precedent—the HMG protein Nhp6a, which plays a role in nucleosome positioning and dynamics at promoters [51],[52], was correlated with the H3R8K mutation (Table S2)—and thus represent potentially novel connections between histone residues and either modifying enzymes or binding partners. Below, we follow up specifically on one such observation, the surprising linkage between H3K4 methylation mutants and the H3S10A histone mutant.

Our data show that joint analysis of histone mutants with related gene deletion mutants can systematically link histone-modifying enzymes with their substrates, as well as modification-specific binding proteins to the relevant modified histone residue (Tables S2 and S3).

### Genome-Wide Histone Modification Dynamics

**Figure 4:**
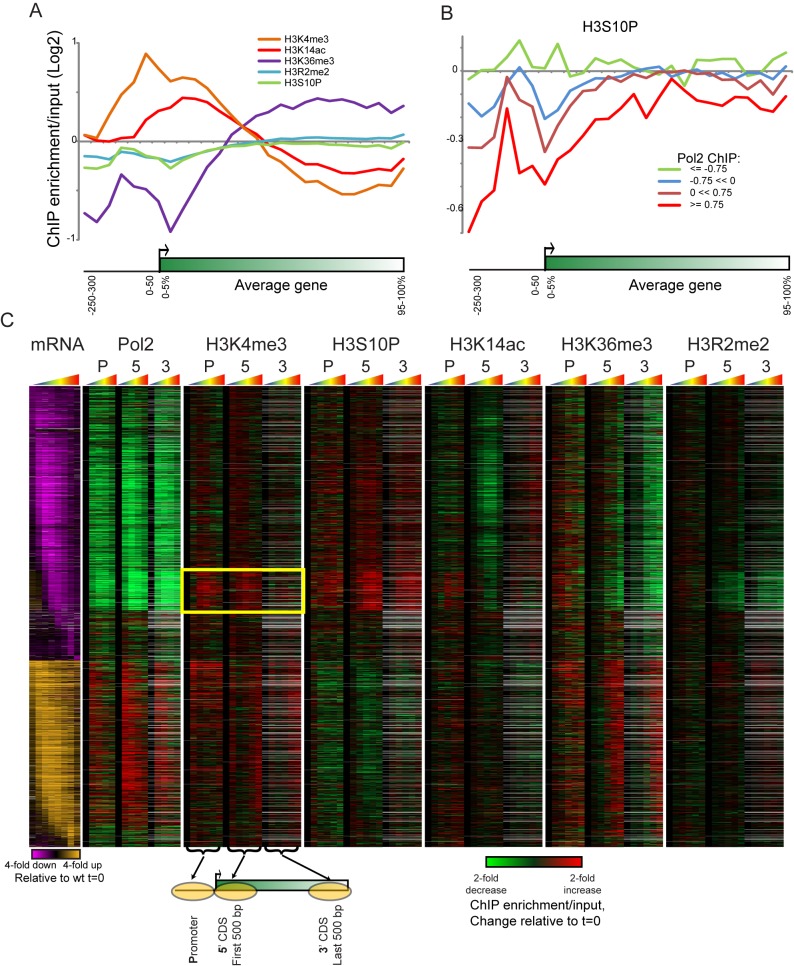
Genome-wide histone modification changes during diamide stress. (A) Metagene analysis of the five histone marks analyzed here. The indicated modifications were mapped genome-wide by ChIP-chip using ∼250 bp resolution tiling microarrays, normalized to nucleosome occupancy. All genes are length-normalized, and ChIP enrichments for the five marks at t = 0 (e.g., midlog growth) are shown averaged for all genes. (B) Metagene analysis for genes grouped according to RNA Polymerase abundance as measured in Kim et al. [26], with H3S10P mapping data averaged for each set of genes. (C) Chromatin changes at all diamide-regulated genes. Genes up- or down-regulated by over 1.8-fold [21] are shown, with mRNA changes represented in orange/purple. Genes are ordered by time of change in gene expression [92]. Tiling microarray probes for Pol2 [26] and for five histone marks were associated with gene promoters, 5′ ends, or 3′ ends as shown in schematic underneath Pol2 panel [31]. Data for each time course are shown as changes relative to t = 0, thus representing the change in the modification over the time course of diamide stress. Pol2 data are for t = 0, 15, 30, 60, and 120 min after diamide stress, whereas histone modification data were collected at t = 0, 4, 8, 15, 30, and 60 min after diamide stress. Grey entries represent missing data (generally due to an absence of any microarray probes at the relevant genomic location). Yellow box highlights “paradoxical” gain of H3K4me3 at the 5′ ends of a large group of diamide-repressed genes.

We next sought to understand why only particular genes were affected by mutants in various chromatin regulators. One of the central questions in chromatin regulation is why broadly localized histone marks appear to have extremely localized effects on gene expression? In other words, given that H3K4me3 occurs at nearly all +1 nucleosomes, why do *set1*Δ mutants exhibit relatively minor [11],[53] gene expression changes? Our functional results suggest that many transcriptional effects of chromatin mutants are masked at steady-state by feedback mechanisms, but can be uncovered during dynamic changes in gene expression. To address the relationship between histone mark occurrence and function in a dynamic context, we therefore extended our studies by carrying out genome-wide mapping of several histone modifications (Tables S4 and S5) during a six time point diamide stress time course (t = 0, 4, 8, 15, 30, and 60 min). We focused these experiments on two relatively well-characterized modifications: H3K36me3 and H3K4me3, and related marks H3K14ac, H3S10P, and H3R2me2a. Our mapping data for unstressed yeast are concordant with known aspects of modification localization patterns from either prior genome-wide mapping efforts [8],[9] or related studies (Figure 4A, Figure S5).

Given the surprising correlation between H3K4A and H3S10A mutants (Figure 3A, Figure S4), we focused on how the histone modifications H3K4me3 and H3S10P change genome-wide during diamide stress. As noted above, H3K4me3 occurs at the 5′ ends of transcribed genes, and genes induced during the stress response gained H3K4me3 over time, as expected (Figure 4C, Figure S5B). H3S10P, which had not been mapped genome-wide in yeast, is most strikingly localized to ∼20 kb surrounding yeast centromeres (Figure S5G), consistent with its pericentric localization by immunofluorescence in mammalian cells [54]. However, we also noted that H3S10P on chromosome arms was heterogeneous, and localized to coding regions with a pattern opposite to that of H3/H4 turnover [31],[35]. H3S10P is depleted from the 5′ ends of genes, and over coding regions anticorrelates with transcription rate (Figure 4B). Furthermore, during the stress response H3S10P levels increase over repressed coding regions, and decrease over induced genes, indicating that the anticorrelation between H3S10P and transcription is dynamic (Figure 4C).

Overall, many of the chromatin changes over stress-activated or repressed genes fit expectations. At stress-activated genes, promoter H3K4me3 levels increased while H3K36me3 increased over gene bodies. However, we also observed several unexpected dynamic behaviors (e.g., increasing H3K36me3 over the promoters of many stress-responsive genes). Furthermore, H3K14, whose acetylation scales with transcription rate during midlog growth [8],[9], was only deacetylated at a small subset of repressed genes during diamide stress, with most repressed genes exhibiting surprisingly minimal changes in H3K14ac (see below).

Most curiously, we found that H3K4me3 levels *increase* at the 5′ ends of a substantial number of diamide-repressed genes during their repression (Figure 4C, yellow box). Not only do these genes gain H3K4me3, they also gain H3S10P, and as noted above H3K4 mutants and H3S10 mutants exhibit similar gene expression defects (Figure 3A, Figure S4). Thus these marks are linked both functionally and in terms of dynamic localization changes. Curiously, the H3K4methylase Set1 and one of the H3S10 kinases, Ipl1, also share the nonhistone substrate Dam1 [55], indicating a more general connection between H3K4 and H3S10 based on shared nonhistone substrates for their modifying enzymes. It is unlikely that the gene expression defects observed here stem from nonhistone substrates of these enzymes as the gene expression changes are observed in histone point mutants as well as modifying enzyme deletions, but the connection is curious nonetheless.

Below, we attempt to connect the changes in H3K4me3 and H3S10P localization with the functional effects of relevant mutants. Are the genes that are misregulated in K4 and S10 mutants the same genes that exhibit dynamic changes in these marks during stress?

### Set1-Dependent H3K4 Methylation Primarily Serves in Gene Repression Rather Than Activation

Set1 methylates H3K4 to create a gradient over coding regions from K4me3 at the 5′ end to K4me1 at the 3′ end, and this methylation pattern correlates with transcription rate during midlog growth ([8],[9], Figure S5B). The correlation between H3K4me3 and transcription rate leads to this mark being referred to as an “activating mark,” yet *set1*Δ mutants exhibit few gene expression defects in midlog growth, and in fact increasing evidence points to a primarily repressive role for K4 methylation in yeast. *set1*Δ mutants exhibit increased basal expression of repressed genes such as *PHO5* [56],[57], and moreover exhibit widespread defects in repression of sense transcription by antisense transcripts [58]–[61].

**Figure 5:**
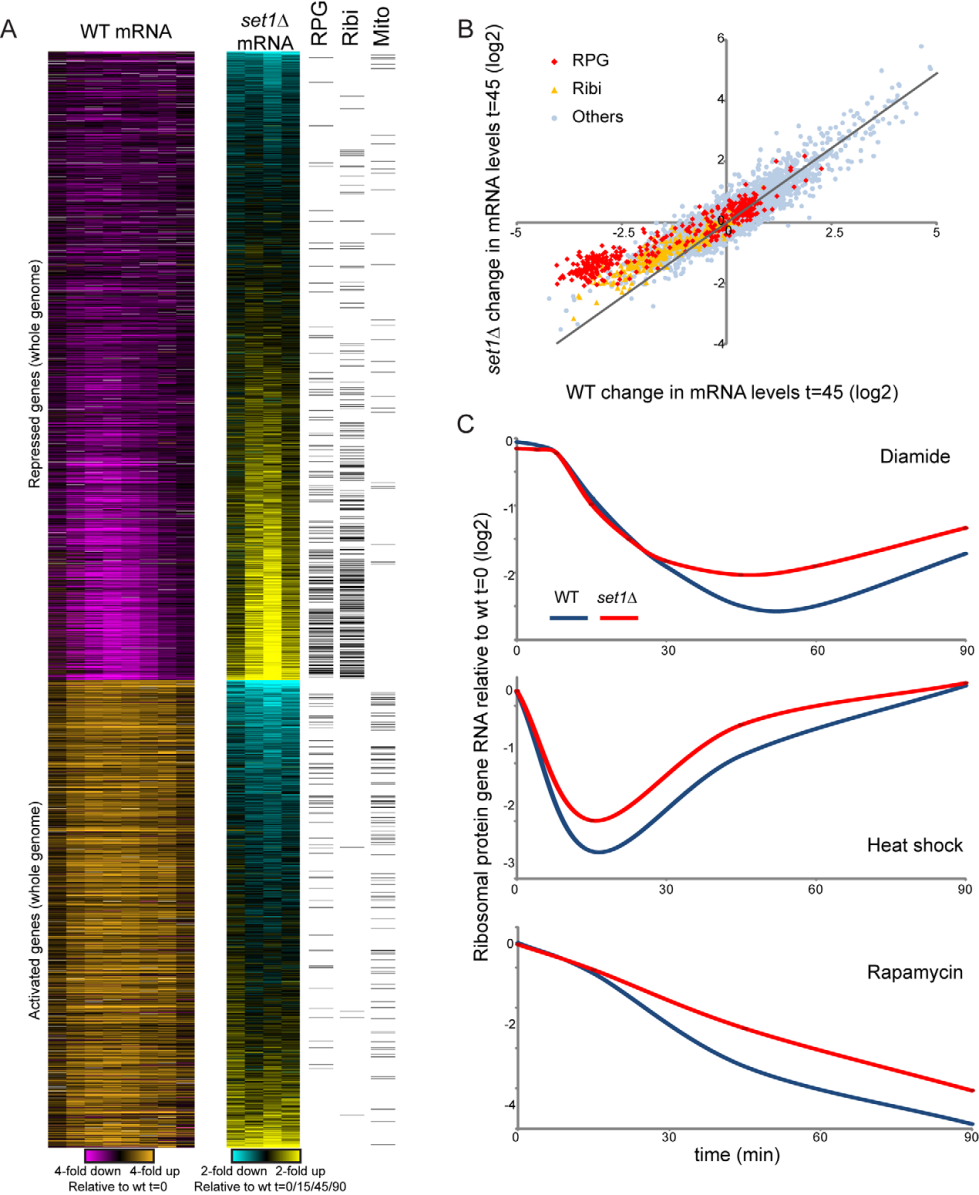
Set1 is predominantly a repressor during diamide stress. (A) Whole genome analysis of *set1*Δ effects on diamide stress response. Left panel: gene expression data from Gasch et al. [21] for all genes induced or repressed at least 1.8-fold. Middle panel: effect of *set1*Δ on diamide stress, for t = 0, 15, 45, or 90 min, using whole-genome microarray data. Genes are grouped by repressed/activated, then subsequently sorted by the average *set1*Δ effect on gene expression. Right panel: ribosomal protein (RPG) or ribosomal biogenesis (Ribi) GO annotations for individual genes are indicated as black bars. The majority of genes that repress poorly in *set1*Δ mutants are involved in cytoplasmic ribosome biosynthesis. (B) Set1 functions primarily as a repressor. Scatterplot of change in mRNA abundance in wild-type (*x*-axis) or *set1*Δ (*y*-axis) yeast at diamide t = 45 min. There is overall excellent correlation between the two datasets except for blunted repression of ribosomal protein genes and ribosomal biogenesis genes, as indicated. (C) Set1 affects ribosomal gene repression in response to multiple distinct environmental conditions. Wild type and *set1*Δ yeast were subjected to time courses of diamide stress, 37°C heat shock, or 0.2 µg/mL rapamycin. RNA abundance was measured by nCounter, and normalized ribosomal protein gene RNA abundances are averaged and shown as log2 abundance. Note that for all three stresses, Set1 is required for full RPG repression.

We noted in our initial gene expression dataset that *set1*Δ and related mutants showed defects in repression of ribosomal protein (“RPG”) and ribosomal biogenesis (“Ribi”) genes (Table S1). We therefore extended these results to whole genome mRNA profiling, finding that the major gene expression defect in *set1*Δ mutants during diamide stress is a failure to adequately repress RPG and Ribi genes (Figure 5A). This result is interesting in light of prior observations that Set1 is required for full repression of the rRNA repeats [62],[63] during steady-state growth (when a subset of rDNA repeats are silenced), and shows that Set1 plays a general role in repression of all aspects of ribosomal biogenesis. Notably, although some snoRNA genes are found in RPG introns, we observed Set1 effects on the majority of ribosomal protein genes, most of which do not carry snoRNAs in their introns, indicating that the observed effect is not a consequence of Set1's known effects on termination at snoRNA genes [64].

Overall, deletion of *SET1* resulted predominantly in diminished repression of ribosome-related genes, with very few large effects on diamide-activated genes (Figure 5B, Figure S6A–C). Importantly, loss of Set1 had a distinct effect on ribosomal gene repression from that observed in “hypo-responsive” mutants. Comparison of a given mutant's effects on overall gene repression to its effects on ribosomal gene repression identifies Set1-related and Sir2-related mutants as having specific defects in ribosomal gene repression (Figure S6D, see also below).

We next asked whether Set1's role in ribosomal repression was specific to diamide stress. We therefore assayed gene expression of our 200 probes in wild type and *set1*Δ yeast responding to another stress response, heat shock, or responding to nutrient deprivation signals induced by the small molecule rapamycin [65],[66]. Each of these stress responses exhibited different repression kinetics of the RPG genes, yet in all three stresses *set1*Δ strains suffered defects in RPG repression (Figure 5C). Thus, Set1 appears to act fairly generally as a repressor of ribosomal biogenesis under suboptimal growth conditions.

### H3K4 Methylation and H3S10 Phosphorylation Jointly Contribute to Ribosomal Protein Gene Repression

**Figure 6:**
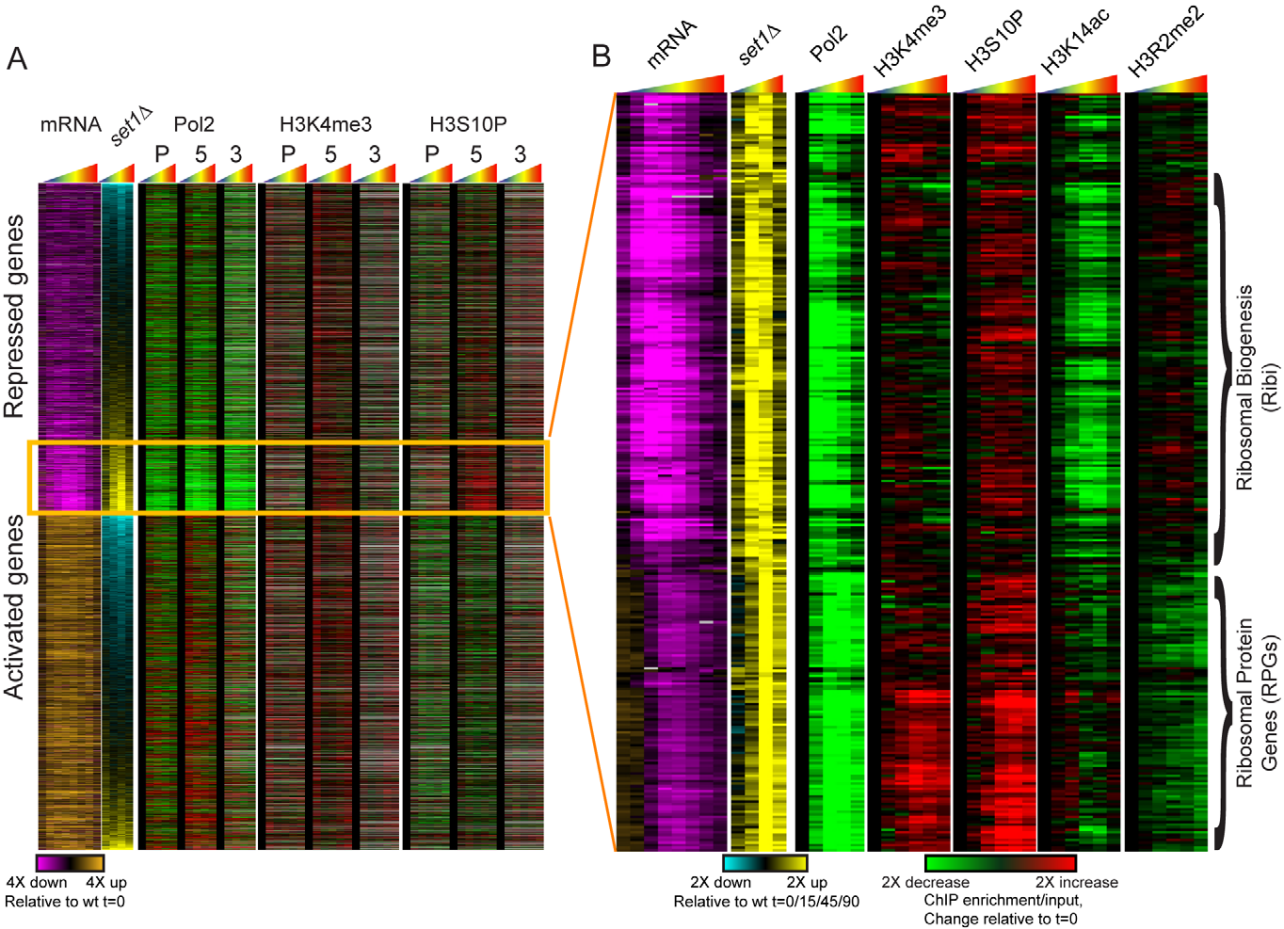
Specific chromatin changes occur at RPG and Ribi genes during repression. (A) Whole genome mRNA [21], *set1*Δ effects on mRNA (this study), Pol2 mapping [26], and tiling microarray data for H3K4me3 and H3S10P (this study) are shown for all genes sorted as in Figure 5A. All four datasets represent a time course of diamide response, as indicated by rainbow triangles above each box. (B) RPG and Ribi genes exhibit distinct chromatin changes during diamide stress. Diamide-repressed genes whose repression is diminished in *set1*Δ mutants were clustered according to their associated changes in chromatin marks. Tiling microarray data are shown only for 5′CDS probes for each mark. A clear separation can be observed between RPGs, which exhibit increased 5′ H3K4me3 and decreased 5′ H3R2me2, and Ribi genes, which exhibit decreased 5′ H3K14ac.

Comparing *set1*Δ effects on mRNA abundance with modification mapping data, we noted that many genes repressed in a Set1-dependent manner were often associated with stress-induced gains in H3K4me3 and H3S10P at their 5′ ends (Figure 6A, Tables S4 and S5). Focusing on the most highly Set1-dependent diamide-repressed genes revealed two clearly distinct clusters based on chromatin changes at the genes' 5′ ends (Figure 6B). Remarkably, we found that ribosomal protein genes (RPGs) were “paradoxically” associated with dramatic gains in H3K4me3 at their 5′ ends, as well as gains in H3S10P. The changes in H3K4me3 and H3S10P were strongest at the +1 nucleosome but occurred throughout the promoters (Figure S7A and analysis not shown). Conversely, non-RPG ribosomal biogenesis (Ribi) genes exhibited similar increases in H3S10P, but modest increases in 5′ H3K4me3. Instead, these genes were among the relatively few diamide-repressed genes associated with decreases in H3K14 acetylation. Importantly, these specific modification changes are quite specific for the gene classes in question. RPGs encompass the majority of genes gaining H3K4me3 during diamide repression, whereas Ribi genes provide the majority of cases with H3K14 deacetylation during repression (Figure S7B–C).

**Figure 7:**
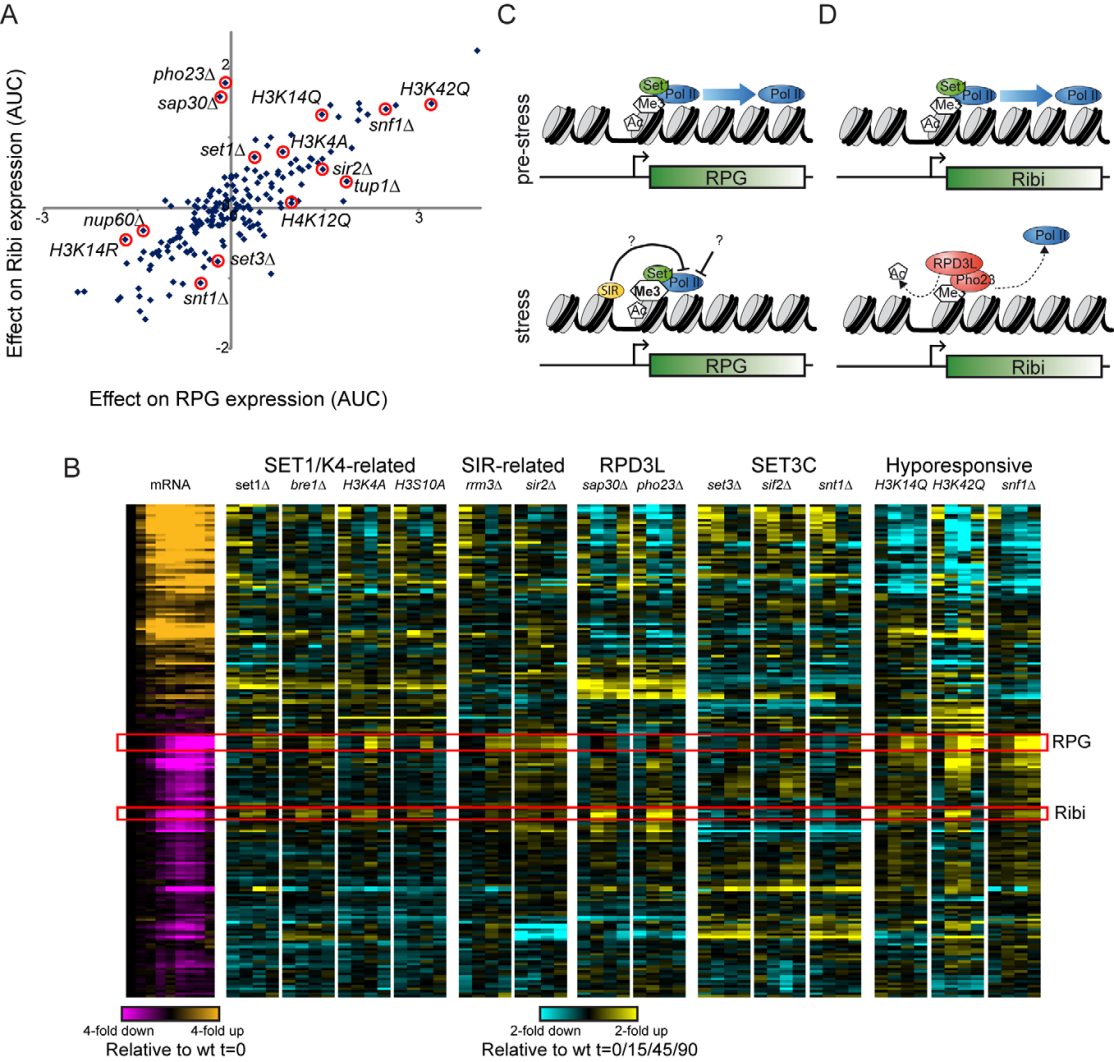
Differential regulation of RPG and Ribi genes by RPD3L. (A) In silico analysis of mutant effects on RPG and Ribi gene expression. nCounter data were averaged for RPG or Ribi genes, and for each mutant the difference between mutant and wild-type expression is scatterplotted for the two gene classes. Specific mutants of interest are indicated with red circles. In general mutants have highly correlated effects on expression of these genes during diamide stress, with globally hypo-responsive mutants such as H3K42Q exhibiting diminished repression of both gene classes. However, a subset of mutants separate RPG from Ribi gene expression. Most notably, mutants in the RPD3L complex (*sap30*Δ and *pho23*Δ) have no effect on RPG repression, but dramatically affect Ribi repression. (B) nCounter data for selected mutants with variable effects on RPG/Ribi gene repression. Data are shown as in Figure 1B,D. Mutants from several complexes of interest are highlighted here. (C–D) Model for Set1-dependent RPG (C) and Ribi (D) repression. See main text.

The distinct chromatin changes observed over RPG and Ribi genes during repression suggested that Set1-dependent repression of these genesets might operate via distinct pathways downstream of H3K4 methylation. We therefore sought to identify additional players in the pathways involved in repression of RPG and Ribi genesets. For each mutant assayed in our nCounter dataset, we compared the effects on diamide repression of RPGs to the effects on Ribi repression (Figure 7A). In general, mutants had similar effects on both gene classes, with globally hypo-responsive mutants such as H3K42Q failing to repress both RPGs and Ribi genes to similar extents. Intriguingly, we found a handful of mutants (several are shown in Figure 7B) with substantially different effects on RPG and Ribi repression: most notably, mutants in the RPD3L complex (e.g., *sap30*Δ, *pho23*Δ) exhibit defective repression of Ribi genes, yet have no effect on RPG gene expression during diamide stress. These results are consistent with prior genome-wide studies in yeast which found that repression of Ribi genes in response to heat shock, H2O2, or rapamycin was defective in the absence of RPD3L [66],[67]. Together, our results suggest that H3K4me3-dependent recruitment or activation of RPD3L (presumably via the PHD finger in Pho23; [57]) is required for Set1-driven repression of Ribi genes, whereas an alternative Set1-dependent pathway, potentially operating via Sir2 (see Discussion), represses RPGs.

Together, these results provide strong evidence for two distinct Set1-dependent gene repression pathways in yeast (Figure 7C–D). Both sets of genes require intact H3K4 and H3S10 for full repression. However, stress-dependent repression of ribosomal biogenesis genes not only requires H3K4 methylation but also is dependent on the RPD3L repressor complex (which likely is recruited to these genes via the PHD finger in Pho23), and these genes specifically are deacetylated during stress. In contrast, repression of ribosomal protein genes is delayed relative to Ribi repression, is largely unaffected by loss of the RPD3L complex, and furthermore these genes are associated with *increased* levels of the “active mark” H3K4me3 during repression.

### Set1-Dependent Regulation of Antisense and Intron-Associated Transcripts

Whereas mutants in our dataset that specifically affect Ribi gene repression suggested a clear mechanistic hypothesis regarding Set1's effects on these genes (H3K4me3-dependent recruitment of RPD3L), we observed relatively few mutants that disproportionately dampened RPG repression relative to Ribi repression. How does H3K4 methylation affect RPG expression? Our first hypothesis, that RPGs could be repressed via H3K4me2-dependent recruitment of the repressive Set3C [58], was ruled out by the observation that mutants in Set3C components do not affect RPG repression (Figure 7A–B).

**Figure 8:**
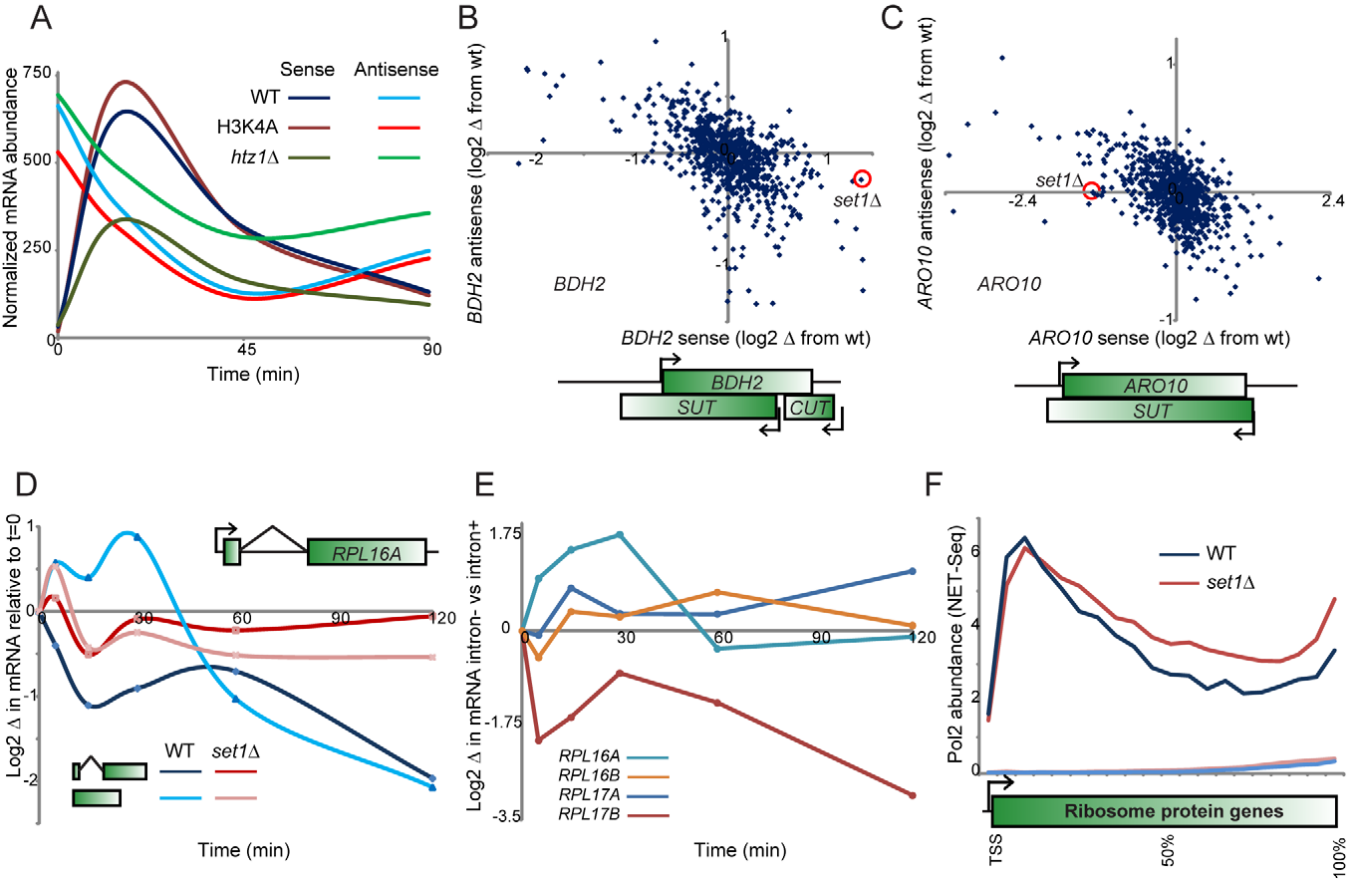
Set1 effects on antisense and intron-containing genes. (A) Anticorrelated abundance of sense and antisense transcripts for *BDH2*. nCounter intensity values are plotted for the sense and antisense transcripts at this locus for wild-type and two indicated mutants. (B–C) Set1 skews sense-antisense ratios. Scatterplot of mutant effects on sense and antisense transcripts for *BDH2* and *ARO10*. Each point in the scatterplot represents the change in expression from wild-type for a given mutant at a specific time point. Here, in both cases, we highlight the effect of *set1*Δ at t = 15 min. For both genes the local transcript structure as defined in Xu et al. [93] is schematized. (D) Regulation of *RPL16A* by its intron. Expression of *RPL16A* was measured by q-RT-PCR (normalized relative to *snR13*), for either wild-type *RPL16A* or for yeast carrying an intronless *RPL16A* in its endogenous location. For both versions of this gene, parallel experiments were carried out in *set1*Δ. (E) Introns contribute to diamide regulation of RPGs. For the four indicated RPGs, the difference in diamide effects on mRNA was calculated for intron-containing versus intronless versions of the gene, as indicated. (F) Set1 plays a role in RPG transcription pausing or termination. Sense strand NET-Seq data from Churchman et al. [84] are shown for all RPGs, for wt and *set1*Δ as indicated. The two lines near the *x*-axis show NET-Seq data on the antisense strand.

An emerging concept in Set1 regulation of yeast genes is that Set1 is required for repression of transcription by trans-acting antisense RNAs [59],[60],[61]. Of the 28 antisense transcripts in our probeset, only a handful were significantly expressed above background during diamide stress. For example, in YPD we find that the *BDH2* sense transcript is expressed at low levels, but its antisense is highly expressed (Figure 8A). Upon diamide treatment, the sense transcript is induced and the antisense is concomitantly repressed. We observed a widespread anticorrelation between mutant effects on sense versus antisense transcripts (Figure 8B). Notably, H3K4 methylation mutants expressed the antisense transcript at lower levels than wild-type in YPD, and conversely hyperinduced the sense transcript during diamide stress. Similar results were observed for the *YTP1* sense/antisense pair (Table S1). In contrast, Set1 had little effect on the level of the antisense transcript at the *ARO10* locus, but instead was required for full induction of the *ARO10* sense transcript in diamide (Figure 8C). Thus, in both cases Set1 primarily affects one transcript in a sense/antisense pair, with the specific transcript being regulated in each case possibly reflecting the fact that the *ARO10* sense does not overlap the TSS of its antisense [61], whereas for *BDH2* the competing transcripts each overlap each other's TSS.

Many of the mutants that affect sense/antisense ratios also affected RPG expression, raising the question of whether expression of these classes of genes might be linked. However, using strand-specific q-RT-PCR we have been unable to find any evidence for antisense transcription of RPGs under our conditions (unpublished data). Instead, based on the curious observation that antisense-mediated repression of *PHO84* in trans requires that the antisense RNA overlap with the *PHO84* UAS [61], we wondered whether some aspect of RNA structure might affect Set1-dependent repression in yeast. Notably, 73% of ribosomal protein genes in yeast carry introns, and these introns are generally much longer than non-RPG introns [68]–[70]. Moreover, RPG introns tend to have more stable secondary structures, both in absolute predicted ΔG of folding and in ΔG per base pair (analysis not shown).

We therefore asked whether RPG introns might contribute to stress-dependent repression of these genes. Figure 8D shows change in expression of *RPL16A*, both for the native gene and for a chromosomally integrated intron-lacking version of *RPL16A*. Notably, diamide repression of this gene was far weaker in the absence of the native intron. We obtained similar results for three of four intronless strains tested, although one intronless gene exhibited hyperrepression in response to diamide stress (Figure 8E). We next asked whether the intronic contribution to *RPL16A* repression was in the same pathway at Set1-mediated H3K4 methylation. As expected from Figure 5, we confirmed that *RPL16A* repression was dramatically diminished in the absence of Set1. Notably, loss of the native intron had little additional effect on repression beyond that observed in the *set1*Δ mutant (Figure 8D), suggesting that Set1-dependent repression of RPGs is somehow connected to their long, potentially highly structured introns. Given the recent observation that RPG introns can affect RNA levels not only of their host genes but also of many paralogs [71], it will be interesting in future studies to determine if such trans-acting gene regulation by introns is Set1-dependent.

## Discussion

We report here a systematic functional genetic analysis of the roles for chromatin regulators and histone mutations in the dynamics of stress response in yeast. We analyzed the effects of 202 chromatin-related mutants on diamide-dependent transcriptional dynamics for 170 RNAs. Importantly, we generalize prior single-gene observations that many chromatin regulators have broader effects on gene induction/repression kinetics than during steady-state growth. Furthermore, we combined these data with whole-genome mapping data for five histone modifications. Together, this dataset provides a rich multidimensional resource for generating hypotheses regarding chromatin biology.

### Chromatin Regulation During Transcriptional Reprogramming

A major observation in this study is that chromatin mutants have far greater effects on gene expression during gene induction/repression than they do on steady-state gene expression in midlog growth. These results are consistent with observations using classic model genes such as *PHO5* and *GAL1-10*, and suggest that a great deal of chromatin biology is obscured at steady-state due to homeostatic mechanisms that compensate for deleted chromatin regulators. These results also suggest that chromatin transitions may often be rate-limiting during transcriptional responses to the environment.

By grouping mutants according to their effects on gene expression, we were able to systematically construct chromatin regulation pathways. These analyses complement similar studies in which deletions are grouped by the similarity of their genetic interaction profiles [14], or according to their gene expression defects in YPD [11]. Importantly, by analysis of gene expression changes during a stress response, we uncover additional interactions that are not observed in YPD. For example, at t = 0 Rph1 and Rpd3 effects on gene expression are highly correlated (*R* ^2^ = 0.51), but during diamide stress they exhibit opposite effects on gene expression (*R* ^2^ = −0.38). These correlations may reflect stress-specific interactions between the factors in question, or they may reflect pathways that operate generally under all conditions but whose effects are only observed during dynamic reprogramming of transcription. Furthermore, by jointly analyzing histone point mutants and deletions of chromatin regulators, we correctly assign many histone-modifying enzymes to their known substrates. We uncover a small number of novel connections here (such as that between Nhp6a and H3R8), but did not find clear connections for predicted histone-modifying enzymes such as Set4. We believe the failure to identify a clear substrate for Set4 likely reflects the low levels of this protein in haploid yeast [72], although we cannot rule out that this enzyme primarily methylates nonhistone substrates, that it functions redundantly with another factor, or other possibilities.

### Joint Analysis of Gene Expression Data and Genome-Wide Mapping Data

As noted in the introduction, the disconnect between global localization of histone marks and their specific, local importance is a key mystery in chromatin at present [73]. Here, we carried out genome-wide histone modification mapping to enable comparisons between the functional effects of chromatin mutants with the locations of relevant marks in a dynamic context.

Overall, our modification mapping data were consistent with extensive prior knowledge about the modifications studied. However, we discovered a number of surprising aspects of histone modification changes during stress responses. For example, we found that H3K36 methylation, typically found over coding regions, was highly dynamic over promoters, suggesting a much more widespread role for this mark in regulation of open reading frames by cryptic transcription [74] than has been previously appreciated. We are currently following up on the role for promoter-localized H3K36me3 in gene regulation. Similarly, while H3K14ac is correlated with transcription rate of genes during steady-state growth (Figure 4, Figure S5; [8],[9]), we found that the majority of genes changing expression in response to diamide stress did not gain or lose H3K14ac in predictable ways. Among repressed genes, deacetylation occurred primarily at genes encoding ribosomal biogenesis factors (Figure S7).

Together, these results highlight the difficulty in understanding the function of specific histone modifications. Clearly, not every gene marked with H3K36me3 requires Set2 for expression. Understanding this phenomenon, often termed “context dependence” of histone modifications, is necessary for a deeper understanding of the biological roles for chromatin regulators [73],[75].

### H3K4 Methylation and Ribosomal Gene Control

Our systematic analyses uncovered several surprising aspects of H3K4 methylation during diamide stress. As noted above, H3K4 methylation is associated with gene transcription at steady-state and thus is considered an “activating” mark, yet in budding yeast most evidence points towards H3K4 methylation as a repressive mark. Loss of Set1 results primarily in derepression of midsporulation and other repressed genes during midlog growth [11],[53],[56],[57]. Set1 appears to broadly play a role in control of sense/antisense ratios [59],[60],[76], as perhaps most clearly demonstrated in the case of the antisense transcript for *PHO84* that is capable of repressing sense transcription in trans [61]. We extend these results, identifying additional sense/antisense pairs regulated by Set1 (Figure 8A–C). It remains unclear, however, what distinguishes sense/antisense pairs subject to Set1 regulation from those that are unaffected by Set1, although in general, transcripts that overlap the promoter of their opposing partner are more likely to regulate the other transcript [61].

Here, we dramatically extend the list of Set1 effects on transcription, finding that during diamide stress Set1 is required for full repression of genes involved in ribosomal biosynthesis. This effect is unlikely to result from nonhistone substrates of Set1, as it is recapitulated in H3K4A mutants. Together with prior observations demonstrating a role for Set1 in rDNA silencing [62],[63] during midlog growth in YPD, our results therefore identify Set1 as a general repressor of ribosomal biogenesis, with roles in repressing rRNA, ribosomal protein genes, and ribosomal biogenesis genes. Importantly, *set1*Δ mutants have no effect on RPG and Ribi gene transcription during active growth in YPD (Figure 5A and [11],[53]), when ribosomal genes are being extremely highly transcribed, meaning that the identification of Set1 as a broad repressor of ribosomal biogenesis could only be observed under stress conditions as in this study. Conversely, since a subset of rDNA repeats are repressed even during active growth, this enabled the discovery of this aspect of Set1 function in early midlog studies.

Based on chromatin mapping and on functional analysis of all 202 mutants, we find that distinct mechanisms operate in the repression of RPGs and the Ribi regulon. Ribi genes, but not RPGs, are not effectively repressed in mutants affecting the RPD3L complex. Moreover, Ribi genes are specifically associated with loss of H3K14ac during diamide stress, but exhibit little to no gain in H3K4me3. These results are consistent with a known pathway in which dephosphorylation of the transcriptional repressors Dot6 and Tod6 leads to RDP3L recruitment to Ribi promoters [67],[77], with binding of RPD3L component Pho23 to H3K4me3 contributing to either RPD3L recruitment or activity [57]. The molecular details underlying the presumptive “bivalent” recruitment/activation of RPD3L by Dot6/Tod6 and H3K4me3 remain to be elucidated. In striking contrast, we find no role for RPD3L in repression of RPGs (Figure 7A–B). Consistent with this, published gene expression profiles from *rpd3*Δ mutants in several stress conditions (diamide was not studied) reveal a far greater effect of Rpd3 loss on repression of Ribi genes than RPGs [67]. This raises the question of how Set1 contributes to RPG repression.

RPG repression was not accompanied by deacetylation of H3K14, and instead we observed that RPG promoters paradoxically *gain* H3K4me3 during diamide repression. It is not immediately apparent what aspect of RPGs makes them subject to Set1-regulated repression, but it is well known that RPGs represent roughly half (102 of 250) of all intron-containing genes in budding yeast. Given the emerging picture that Set1 affects gene regulation by antisense RNAs associated with promoters, we speculated that ribosomal introns and promoter-associated antisenses might share in common some unusual form of locally tethered RNA secondary structure. Ribosomal introns are longer than most other introns in yeast, and generally have much greater predicted RNA secondary structure than other introns. Consistent with the idea that RPG introns might contribute to Set1-dependent repression, we found that in several cases replacement of the native intron-containing RPG with its cDNA (in the native chromosomal context) abrogated repression of the RPG by diamide (Figure 8D–E), suggesting that either the intronic RNA or the corresponding DNA plays a role in Set1-dependent repression of some RPGs. As for the downstream repressor, we are currently investigating the hypothesis that RPG repression could be mediated by the Sir heterochromatin complex. Genome-wide mapping studies show that Sir3 binds to RPGs [78]–[80], and we show here that *sir* mutants and *set1* mutants have similar effects on RPG repression (Figure 7B). Moreover, in vivo selection studies for RNA-based repressors in yeast found a surprisingly high fraction of tethered RNAs could repress a reporter gene in a Sir-dependent manner [81], suggesting that structured RNAs might recruit the Sir complex in a manner analogous to the role for lincRNAs in repressing metazoan genes by Polycomb recruitment [82],[83]. In this view, we hypothesize that ribosomal introns might serve in some sense as “domesticated” lincRNAs.

Alternative hypotheses include the possibility that the act of splicing per se could play a role in Set1-dependent repression of RPGs (whose splicing is mechanistically distinct from non-RPG splicing; [69],[70]) or that Set1 affects RPG expression by regulating Nrd1-dependent transcriptional termination [64]. Intriguingly, we observed in published NET-Seq data on Pol2 localization [84] that *set1*Δ mutants exhibit lower 5′ peaks of Pol2 over RPGs during midlog growth, with increased Pol2 levels downstream (Figure 8F). This decrease in 5′ Pol2 localization is consistent with the possibility that Set1 regulates RPGs via effects on transcriptional termination. It is also consistent with an alternative mechanism in which Set1 regulates Pol2 pausing at the 5′ ends of RPGs and that the delayed Pol2 in wild-type cells either allows intron folding or simply keeps 5′ RNA physically tethered near the promoter.

Future studies will be required to determine whether RPGs and antisense-regulated genes do in fact operate via a common mechanism, and to identify whether any specific aspects of RNA or RNA/DNA structures play a role in recruiting repressive complexes.

### Conclusion

Taken together, these data show that chromatin regulators have far more effects on changes in gene expression than on steady-state transcription. Our approach allows systematic linking of chromatin regulators in complexes and of histone-modifying enzymes with their substrates. Finally, we show that joint analysis of functional gene expression data with localization data leads to novel insights even into extensively studied histone modifications such as H3K4me3.

## Materials and Methods

### Yeast Strains and Growth Conditions

Two collections of yeast mutants were used. Histone point mutants were described in [27], and were a kind gift from Jef Boeke. Diploid heterozygous deletion mutants with the SGA reporter developed by [85] were sporulated and selected to generate haploid Mat**a** knockouts [86]. Yeast knockout mutants were grown on selective media (SC–Leu–His–Arg dropout mix+G418 200 mg/L+ L-Canavanine 6 mg/L) for two rounds to select for the deletion and for haploids, then used in the nCounter assays.

For Nanostring nCounter assays, each strain was grown in 80 mL YPD to mid-log phase (OD600 between 0.4 and 0.6) in a shaking 30°C waterbath. At “time zero” cells were treated with 1.5 mM diamide (D3648, Sigma), and 3 mL samples of culture each were taken at t = 0 (immediately prior to diamide addition), 4, 8, 15, 22.5, 30, 45, 60, and 90 min. Samples were immediately fixed with 4.5 mL cold (−45°C) methanol and kept in dry ice-ethanol bath throughout the time course. Cells in each sample were pelleted at 4,000 rpm for 2 min at 4°C, washed with 10 ml nuclease-free water, resuspended in 1 mL RNAlater solution (Ambion), and stored at −80°C.

For the histone modification mapping time course, six flasks each of 400 mL BY4741 cells were grown in YPD to mid-log phase shaking at 220 rpm at 30°C. Cells were treated with 1.5 mM diamide at time zero. At t = 0, 4, 8, 15, 30, and 60 min, cells were fixed by 1% formaldehyde, followed after 15 min by quenching with 125 mM glycine. Cells were then pelleted, washed with water, and subjected to MNase digestion as previously described [33],[87] and immunoprecipitation (see below).

### nCounter Assays (Nanostring Technologies, Seattle, WA)

Approximately 1×10^7^ cells from individual samples were pelleted and resuspended in 600 µL Qiagen RLT buffer. After bead beating for 3 min, the supernatants were collected and 3–5 µL of the cell extracts were used for nCounter assays. The nCounter assays were performed as described [25] with customized probes corresponding to 200 *S. cerevisiae* RNAs.

### nCounter Data Normalization

The nCounter dataset reports on the measurement of 200 probes×202 mutants×4 time-points. We denote by *M_i,j_*, the measurement of probe 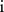 sample 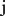. To account for differences in hybridization, processing, binding efficiency, and other experimental variables, we used to following normalization procedure:

1. Each sample was normalized relative to the average of four *wild-type* replicates taken at the same time point after diamide induction. First, samples were log transformed. Next, we assumed that different samples (WT versus mutant) could be brought on to the same scale by a linear regression (assuming that in the same time point most of the genes do not change their expression level). This was parameterized by two real values *b_j_* and *a_j_*>0 corresponding to background subtraction (*b_j_*) and global normalization factor (*a_j_*). Specifically, *a_j_* is a multiplicative factor that is used to control the assay efficiency or to bring the total RNA counts roughly to the same levels, and *b_j_* is an additive factor that corresponds to the average background counts of each sample. We estimated the values of these two normalization parameters for each sample using linear regression and normalized the data using the following equation:
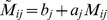

2. To overcome the limitation of the log-ratio statistics for weakly expressed genes (increase from 50 to 100 reads is not as significant as the increase from 100,000 to 200,000 reads), we used the variance stabilization method as described in [88]. Briefly, this involves estimating a statistic Δ*h* whose variance is approximately constant along the whole measurement scale. For highly expressed genes, Δ*h* and the log-ratio statistic coincide. We estimated parameters of the statistic Δ*h* as described by Huber et al., and represented the data as pseudo-log likelihood to WT at time the matching time point (Figure 1, Table S1).

### Correlation Analysis of Expression-Profiles

We computed a correlation matrix (Figure 3A) by first concatenating the measurements (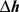 values) for all probes at the four time points to a single vector for each mutant, and then computing the Pearson correlation between the vectors for each pair of mutants. We clustered the correlation matrix using hierarchical clustering with Euclidian distance metric and unweighted average distance (UPGMA) linkage. Clustering was done using MATLAB 7.10 procedures “pdist,” “linkage,” and “dendrogram.”

To identify significant correlations between mutants we used a quantile-quantile plot. For a query mutant, we plotted the quantiles of its correlations vector with all other mutants versus theoretical quantiles from a normal distribution (function “qqplot,” MATLAB 7.10). Values that deviate from the line y = x were considered significant (Figure S4).

### PCA Analysis

Principal component analysis was applied to the map of 200 probes versus 202 mutants using MATLAB 7.10 procedure “princomp.”

### Microscopy

To evaluate transcriptional induction in individual cells in a population, we performed time-lapse microscopy of the induction of GFP-tagged protein. Nine deletion strains (*hda2*Δ, *yta7*Δ, *spt8*Δ, *set1*Δ, *rph1*Δ, *snf1*Δ, *cac2*Δ, and *swc3*Δ) were generated using KanMX in the BY4742 background. Four GFP-fusion reporters were selected (*GCY1*, *GRE3*, *PGM2*, and *TSA2*) from a library (Breker and Schuldiner, personal communication) based on the yeast GFP-tagging library [89] with an additional constitutive cytoplasmic mCherry (Genotype: xxx-GFP::HIS3, p*TEF2*-cherry::*URA3*, *his3* 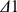 *leu2* 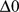 met15
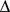
0 *ura3* 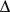
0 *lyp1* 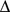 *can1* 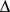::p*MFA1*-*LEU2*). Knockout strains were mated with GFP reporter strains and sporulated to generate haploid deletions carrying the GFP reporters.

Prior to assay, strains were grown in 96-well plates to mid-log (∼0.6 OD 600) in synthetic complete media (SC). We then transferred cells to glass bottom microwell plates (384 format, Matrical Biosciences) pre-treated with concavalin-A (incubation with solution at 0.25 mg/ml for 15 min). Cells were allowed to settle onto the glass surface for 30 min. We then removed the media and replaced with treatment media (SC with 1.5 mM diamide).

Following induction we placed the cell on an automated microscope (Scan∧R system, Olympus) and assayed with 40× objective at ∼10 min intervals, taking transmitted light, mCherry, and GFP images at each time point. Images were analyzed using custom-made software, written in python based on the OpenCV image analysis package (http://opencv.willowgarage.com/). Briefly, the procedure detects cells by thresholding the mCherry image and finding contours of bright objects. Contours that meet gating criteria for circularity and size were considered cells. The procedure matched detected cells in successive images based on a reciprocal closest hit procedure allowing a maximum of 5 pixel movement. Since cells are adhered to the glass surface, this procedure was effective in following a single cell. If there was a budding event, the closest-hit procedure returns an ambiguous result and the match is not made. Cells that were traced throughout the time course were used in the further analysis steps.

### Model

We represented each single cell time-course GFP measurements using a simple kinetic model. We assume that the transcription starts at a certain point following stimuli, termed *t_on_*, and stops at *t_off_*. Promoter behavior is represented by *T*(*t*):


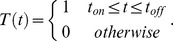


Only during this time interval is mRNA being transcribed; to simplify the model we assume that transcription occurs with a constant rate 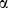 of mRNA/min, and we also assume a constant exponential decay rate of mRNA denoted by 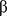. We present the mRNA levels as a function of time using the following differential equation:


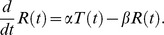


Solving this equation we obtained a logistic equation describing mRNA level over time after stress induction at t = 0:


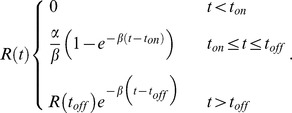


In the final step we assume that protein is the integration of the mRNA levels (assuming constant translation rate without degradation). We add a final parameter to account for the basal GFP level (prior to stress), solved the integral of 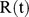, and obtained the following equation:


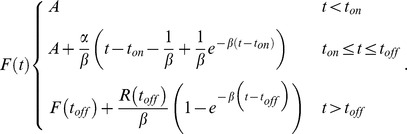


To estimate the parameters for each single cell track, we used MATLAB 7.10 function “fmincon” using the “active-set” optimization algorithm.

#### Chromatin immunoprecipitation (ChIP)

ChIP assays were carried out as previously described [87]. Antibodies used in the ChIP assays were anti-H3K36me3 (ab9050-100, Lot#412997, Abcam), anit-H3K4me3 (04-745, Lot#NG1643014, Millipore), anti-H3K14ac (07-353, Lot#DAM1548623, Millipore), anti-H3S10p (04-817, Lot#NG1710274, Millipore), and anti-H3R2me2 (07-585, Lot#DAM1731499, Millipore).

### Microarray Hybridization of ChIP Material

ChIP material was amplified using the DNA linear amplification method described previously [8],[90]. 3 ug of the amplified ChIP products was labeled via the amino-allyl methods as described on http://www.microarrays.org. Labeled probes (a mixture of Cy5-labeled input and Cy3-labeled ChIP-ed material) were hybridized onto an Agilent yeast tiled oligonucleotide microarray (G4495A) at 65°C for 16 h and washed as described on http://www.microarrays.org. The arrays were scanned at 5 µ resolution with an Agilent scanner. Image analysis and data normalization were performed using Agilent feature extraction.

## Supporting Information

Figure S1 Overview of experimental and analytical approach. (A) For each of 202 mutants analyzed, mutant was grown to midlog, then treated with diamide to induce a transcriptional stress response. (B) Expression of 200 transcripts was analyzed by nCounter analysis, and for each mutant gene expression defects relative to wild-type were calculated. (C) Mutants with similar gene expression profiles were clustered, identifying chromatin regulatory complexes and connections between chromatin regulators and specific histone residues. (D) In parallel, wild-type yeast were treated with diamide for a time course and five histone modifications were mapped genome-wide using tiling microarrays. (E) Diamide-regulated genes were clustered to identify patterns of histone modifications over specific subsets of induced or repressed genes. (F) Combining functional data with localization data lead to a number of mechanistic hypotheses, one of which we investigated in greater detail.
(TIF)

Figure S2 Hypo- and hyper-responsive mutants. (A) Data from Figure 1 sorted by the effect of each mutant on transcriptional induction/repression. Mutants to the right represent “hyporesponsive” mutants exhibiting a blunted diamide stress response. (B) Representation of the first principal component of the nCounter dataset. The relative contribution of only the first principal component of the entire dataset is shown here in blue-yellow heatmap, with mutants and genes ordered as in (A). Right panel shows whether genes are regulated primarily by TFIID or by SAGA [94]. (C) Responsiveness to chromatin mutants correlates with promoter nucleosome occupancy. The *x*-axis shows the effects of hyper/hyporesponsive mutants on diamide regulation of each probe in our dataset, and the *y*-axis shows average nucleosome occupancy [30] for 500 bp upstream. Genes are colored red-green based on their induction/repression in wild-type. (D) Hyporesponsive histone point mutants occur at histone-DNA contact areas. Mutations exhibiting diminished amplitude of the diamide stress response are mapped on to the nucleosome crystal structure and are shown as sphere models in red. All mutants in the globular domains of H3 and H4 occur at histone-DNA contact regions.
(TIF)

Figure S3 Comparison of K→R and K→Q mutations. Correlation between K→R and K→Q mutations for 23 H3/H4 lysines. R and Q mutations exhibit high correlations for several lysines whose modified state has a well-understood binding partner (e.g., H3K36me3-Eaf3, H4K16ac-Sir3). Conversely, residues for which R and Q mutations had anticorrelated effects on gene expression were generally known acetylation sites (and showed similar effects to deletion mutants in histone acetylases and deacetylases, as indicated), and could plausibly report on charge-dependent chromatin transactions.
(TIF)

Figure S4 Identification of chromatin pathways from correlation matrix. (A) Identification of significant associations between mutants. QQ plot shows, for each mutant, the correlation with the test mutant (here, *set2*Δ) on the *x*-axis, with the theoretical distribution of correlations expected from a normal distribution on the *y*-axis. Points distant from the x = y line are significant correlations. (B) Local cluster of mutants significantly correlated with *set2*Δ. Data from Figure 3A, re-clustered using only highly-correlated mutants with *set2*Δ. (C–D) As in (A–B), but for H3K4A. (E–F) As in (A–B), for *sir2*Δ.
(TIF)

Figure S5 Genome-wide histone modification mapping. (A) Genome-wide mononucleosome-resolution mapping of H3K4me3, H3K36me3, H3S10P, H3K14ac, and H3R2me2 was carried out by ChIP-chip (relative to mononucleosomal input to control for histone occupancy) using ∼265 bp resolution tiling microarrays. All open reading frames are converted to a “metagene” with six bins reporting on 50 bp increments from 0 to 300 bp upstream of the TSS and 20 bins reporting on 5% intervals covering the ORF. (B–F) Genes are broken into four classes according to Pol2 levels [26], and data for the indicated modifications are presented as in (A). (G) H3S10P localization to pericentric regions. All chromosomes are aligned by their centromere, and H3S10P mapping data are shown in red-green heatmap.
(TIF)

Figure S6 Set1 is a ribosomal repressor during stress. (A) Histograms of *set1*Δ effects on genome-wide diamide-induced gene expression. Histograms are shown for all genes, for genes unaffected by diamide (<1.8-fold change in expression) or up- or down-regulated >1.8-fold by diamide, as indicated. Note that only repressed genes show substantially different behavior than the bulk behavior of all genes, with very few activated genes showing Set1 dependence. (B–C) As in (A), but for each individual time point of diamide treatment. Genes are broken into activated (B) and repressed (C) based on their maximal fold change during the time course. (D) Set1 is not a general hyporesponsive mutant. For all mutants analyzed by nCounter, the average effect on repressed genes (*x*-axis) is plotted against the effect specifically on RPG repression (*y*-axis). Overall effect on repression is calculated as the area under the curve (AUC) across the entire time course. General hyporesponsive mutants are found in the upper right quadrant, while mutants related to H3K4 methylation or the Sir complex are located above the diagonal, indicating specific defects in ribosomal gene repression.
(TIF)

Figure S7 Specific chromatin changes associated with RPG and Ribi repression. (A) Specificity of H3K4me3 gain at RPG 5′ ends. Probes corresponding to the +1 nucleosome at RPGs were analyzed specifically, and time course data for all five modifications are shown as indicated. (B) The majority of diamide-repressed genes that gain H3K4me3 are RPGs. Diamide-repressed genes are sorted by the 5′ change in H3K4me3, with GO annotations shown in the right panel as indicated. (C) The majority of diamide-repressed genes that lose 5′ H3K14ac are Ribi genes. As in (B), but with genes sorted by 5′ change in H3K14ac.
(TIF)

Table S1 Gene expression data. All data for Figure 1E–F.
(XLSX)

Table S2 Correlation matrix between mutant effects on gene expression. Data for Figure 3A.
(XLSX)

Table S3 Significant correlations between mutants. Data used for Figure 3B.
(XLSX)

Table S4 Modification mapping data part I. Agilent tiling microarray data for H3K36me3 and H3K14ac at 6 time points during a diamide stress.
(XLSX)

Table S5 Modification mapping data part II. Agilent tiling microarray data for H3K4me3 and H3S10P at 6 time points during a diamide stress. These data and data for H3R2me2 also available at at GEO, accession# GSE39080.
(XLSX)

## Abbreviations

ChIP: chromatin immunoprecipitation
GFP: green fluorescent protein
Ribi: ribosomal biogenesis
RPG: ribosomal protein gene
rRNA: ribosomal RNA
