# Supplementary figures and images for: Systematic Dissection of Roles for Chromatin Regulators in a Yeast Stress Response
Source: PLoS Biol. 2012 Jul 31;10(7):e1001369. doi: 10.1371/journal.pbio.1001369 (PMC3416867; doi:10.1371/journal.pbio.1001369)

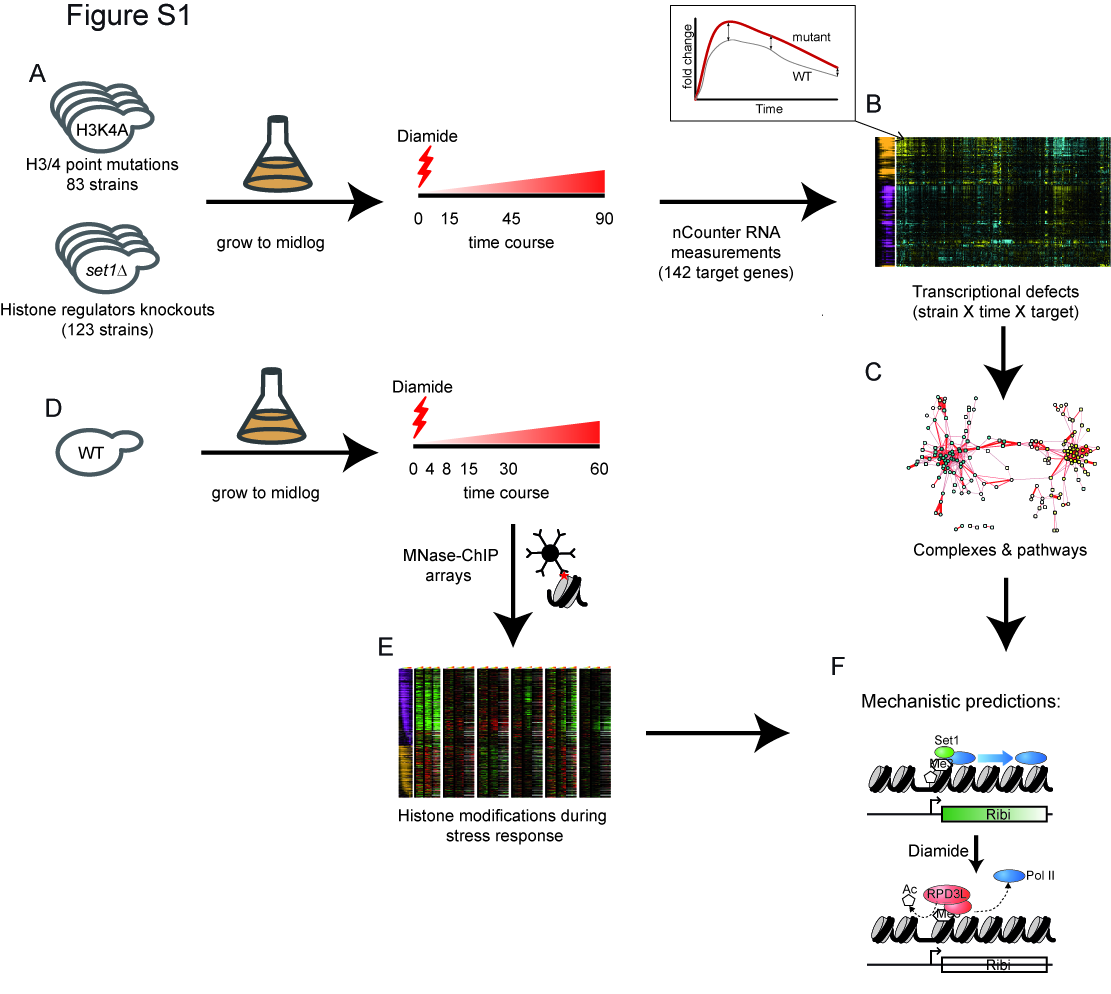

Supplement: Figure S1 — Overview of experimental and analytical approach. (A) For each of 202 mutants analyzed, mutant was grown to midlog, then treated with diamide to induce a transcriptional stress response. (B) Expression of 200 transcripts was analyzed by nCounter analysis, and for each mutant gene expression defects relative to wild-type were calculated. (C) Mutants with similar gene expression profiles were clustered, identifying chromatin regulatory complexes and connections between chromatin regulators and specific histone residues. (D) In parallel, wild-type yeast were treated with diamide for a time course and five histone modifications were mapped genome-wide using tiling microarrays. (E) Diamide-regulated genes were clustered to identify patterns of histone modifications over specific subsets of induced or repressed genes. (F) Combining functional data with localization data lead to a number of mechanistic hypotheses, one of which we investigated in greater detail. (TIF) [file pbio.1001369.s001.tif]

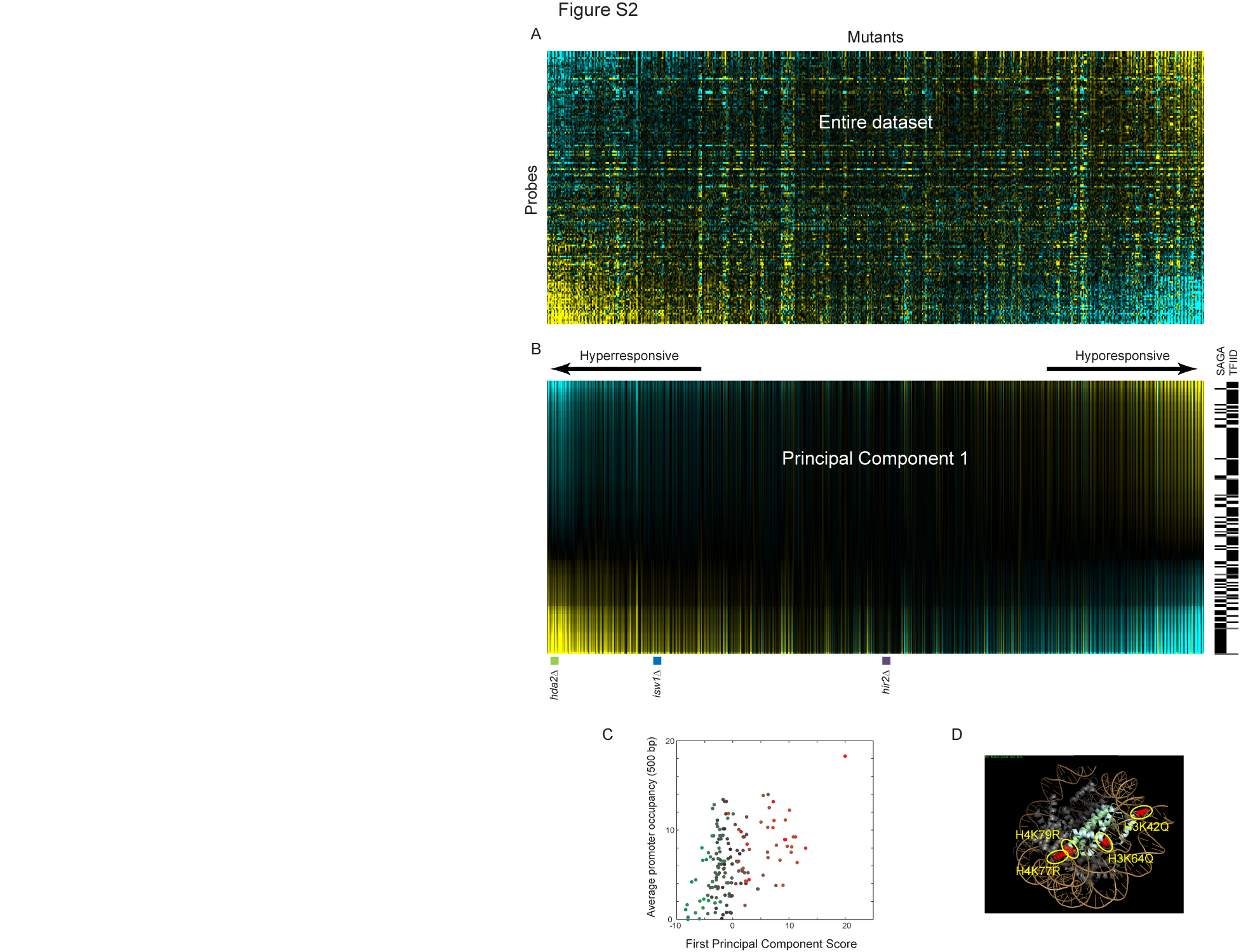

Supplement: Figure S2 — Hypo- and hyper-responsive mutants. (A) Data from Figure 1 sorted by the effect of each mutant on transcriptional induction/repression. Mutants to the right represent “hyporesponsive” mutants exhibiting a blunted diamide stress response. (B) Representation of the first principal component of the nCounter dataset. The relative contribution of only the first principal component of the entire dataset is shown here in blue-yellow heatmap, with mutants and genes ordered as in (A). Right panel shows whether genes are regulated primarily by TFIID or by SAGA [94]. (C) Responsiveness to chromatin mutants correlates with promoter nucleosome occupancy. The x-axis shows the effects of hyper/hyporesponsive mutants on diamide regulation of each probe in our dataset, and the y-axis shows average nucleosome occupancy [30] for 500 bp upstream. Genes are colored red-green based on their induction/repression in wild-type. (D) Hyporesponsive histone point mutants occur at histone-DNA contact areas. Mutations exhibiting diminished amplitude of the diamide stress response are mapped on to the nucleosome crystal structure and are shown as sphere models in red. All mutants in the globular domains of H3 and H4 occur at histone-DNA contact regions. (TIF) [file pbio.1001369.s002.tif]

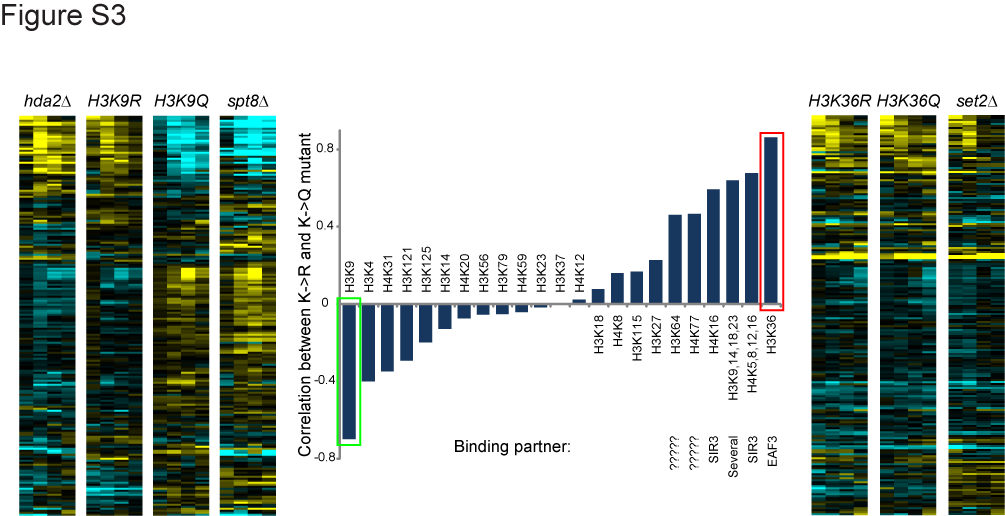

Supplement: Figure S3 — Comparison of K→R and K→Q mutations. Correlation between K→R and K→Q mutations for 23 H3/H4 lysines. R and Q mutations exhibit high correlations for several lysines whose modified state has a well-understood binding partner (e.g., H3K36me3-Eaf3, H4K16ac-Sir3). Conversely, residues for which R and Q mutations had anticorrelated effects on gene expression were generally known acetylation sites (and showed similar effects to deletion mutants in histone acetylases and deacetylases, as indicated), and could plausibly report on charge-dependent chromatin transactions. (TIF) [file pbio.1001369.s003.tif]

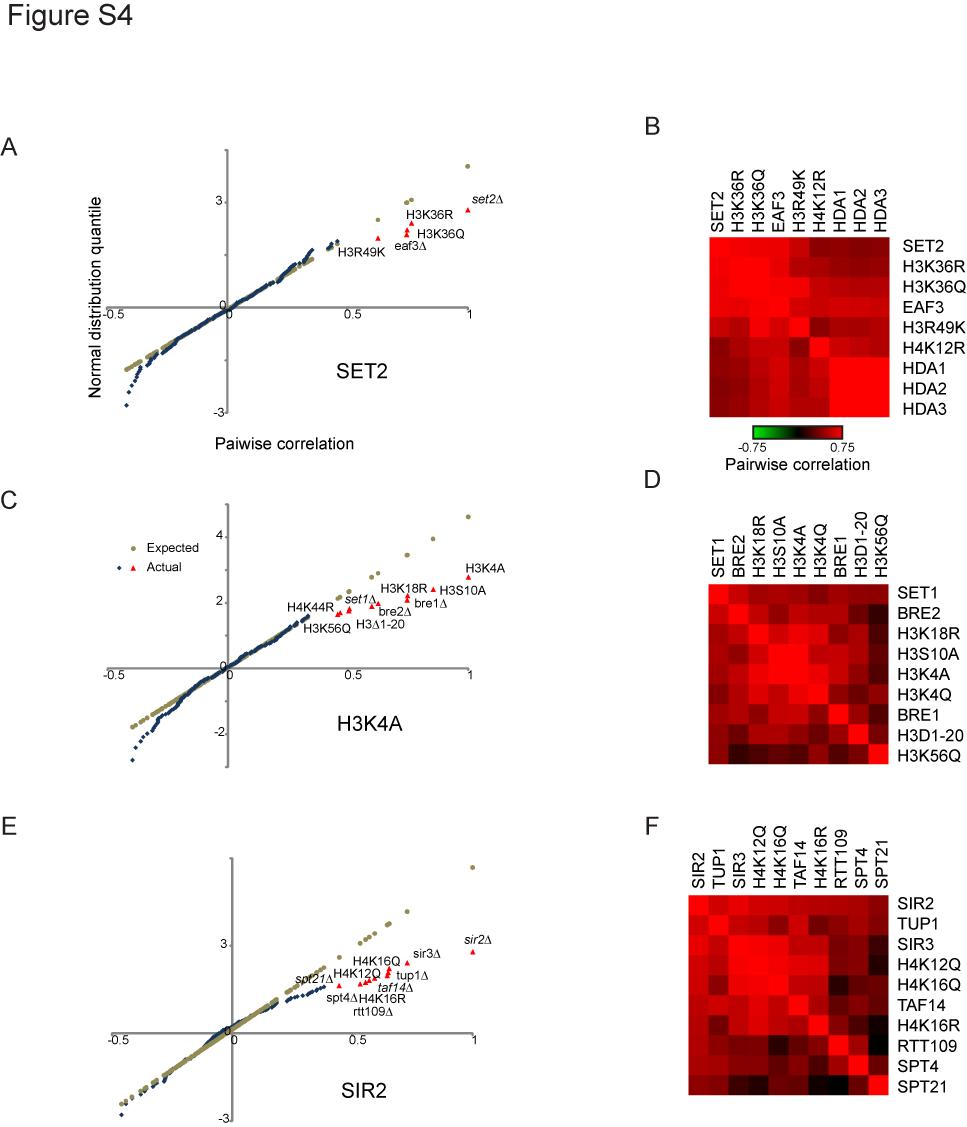

Supplement: Figure S4 — Identification of chromatin pathways from correlation matrix. (A) Identification of significant associations between mutants. QQ plot shows, for each mutant, the correlation with the test mutant (here, set2Δ) on the x-axis, with the theoretical distribution of correlations expected from a normal distribution on the y-axis. Points distant from the x = y line are significant correlations. (B) Local cluster of mutants significantly correlated with set2Δ. Data from Figure 3A, re-clustered using only highly-correlated mutants with set2Δ. (C–D) As in (A–B), but for H3K4A. (E–F) As in (A–B), for sir2Δ. (TIF) [file pbio.1001369.s004.tif]

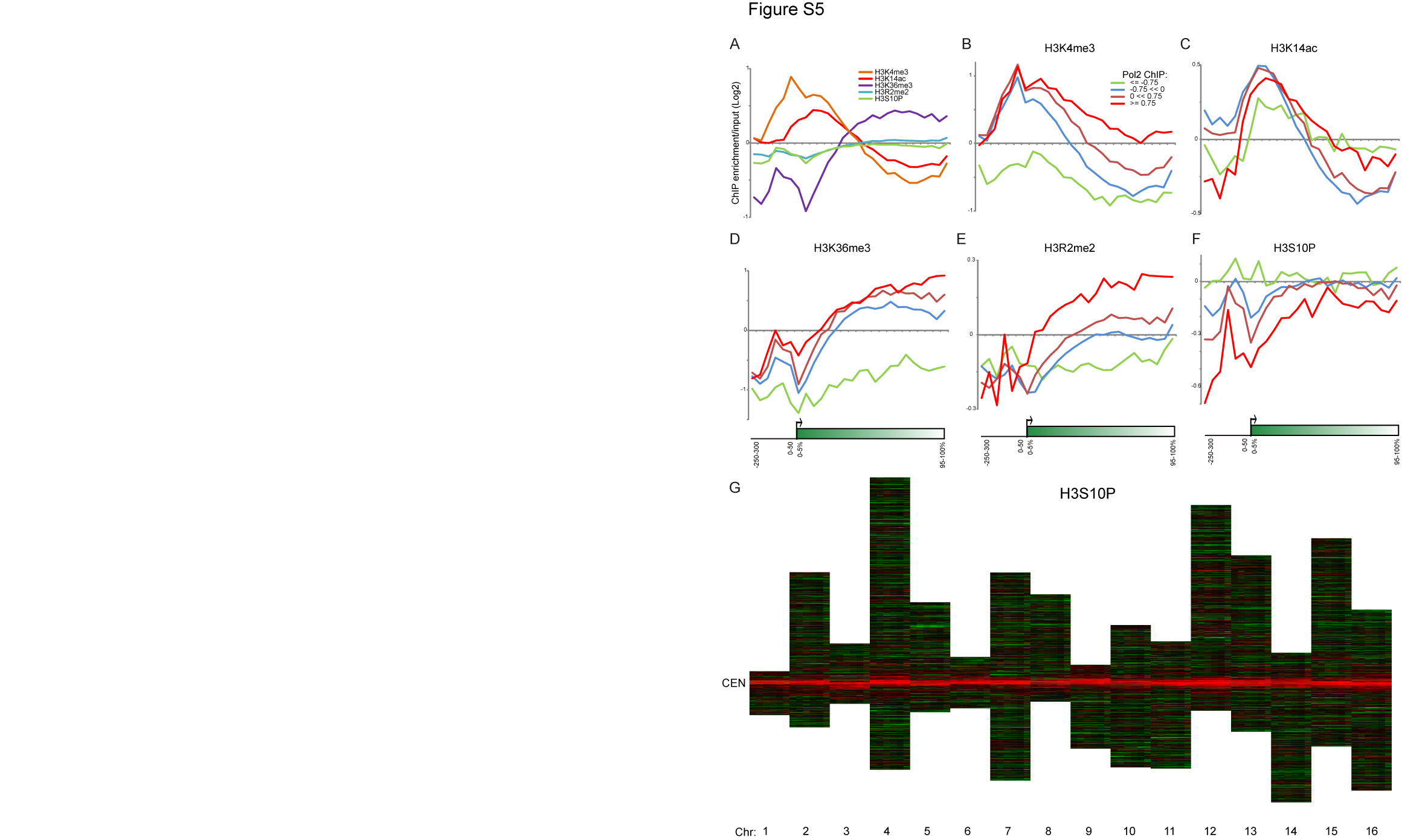

Supplement: Figure S5 — Genome-wide histone modification mapping. (A) Genome-wide mononucleosome-resolution mapping of H3K4me3, H3K36me3, H3S10P, H3K14ac, and H3R2me2 was carried out by ChIP-chip (relative to mononucleosomal input to control for histone occupancy) using ∼265 bp resolution tiling microarrays. All open reading frames are converted to a “metagene” with six bins reporting on 50 bp increments from 0 to 300 bp upstream of the TSS and 20 bins reporting on 5% intervals covering the ORF. (B–F) Genes are broken into four classes according to Pol2 levels [26], and data for the indicated modifications are presented as in (A). (G) H3S10P localization to pericentric regions. All chromosomes are aligned by their centromere, and H3S10P mapping data are shown in red-green heatmap. (TIF) [file pbio.1001369.s005.tif]

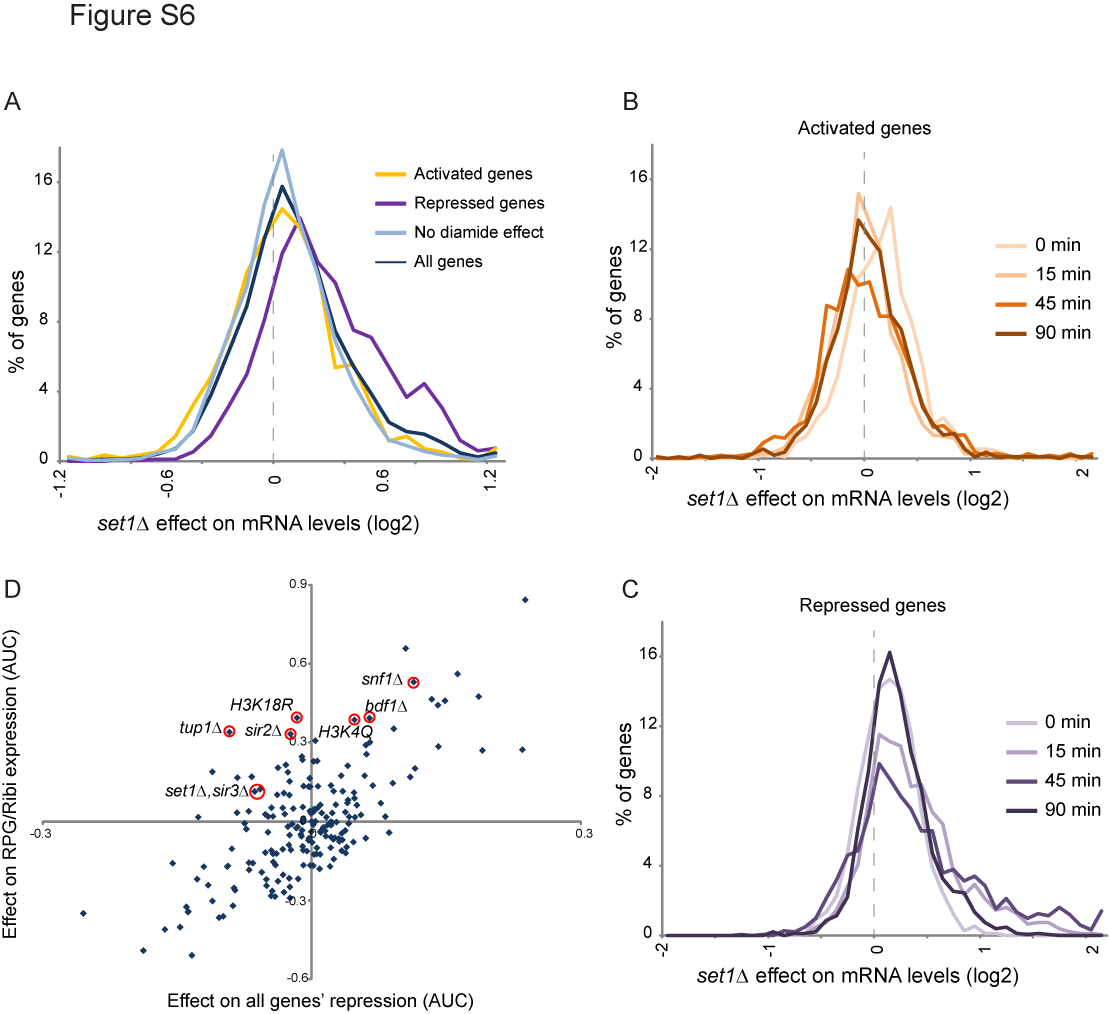

Supplement: Figure S6 — Set1 is a ribosomal repressor during stress. (A) Histograms of set1Δ effects on genome-wide diamide-induced gene expression. Histograms are shown for all genes, for genes unaffected by diamide (<1.8-fold change in expression) or up- or down-regulated >1.8-fold by diamide, as indicated. Note that only repressed genes show substantially different behavior than the bulk behavior of all genes, with very few activated genes showing Set1 dependence. (B–C) As in (A), but for each individual time point of diamide treatment. Genes are broken into activated (B) and repressed (C) based on their maximal fold change during the time course. (D) Set1 is not a general hyporesponsive mutant. For all mutants analyzed by nCounter, the average effect on repressed genes (x-axis) is plotted against the effect specifically on RPG repression (y-axis). Overall effect on repression is calculated as the area under the curve (AUC) across the entire time course. General hyporesponsive mutants are found in the upper right quadrant, while mutants related to H3K4 methylation or the Sir complex are located above the diagonal, indicating specific defects in ribosomal gene repression. (TIF) [file pbio.1001369.s006.tif]

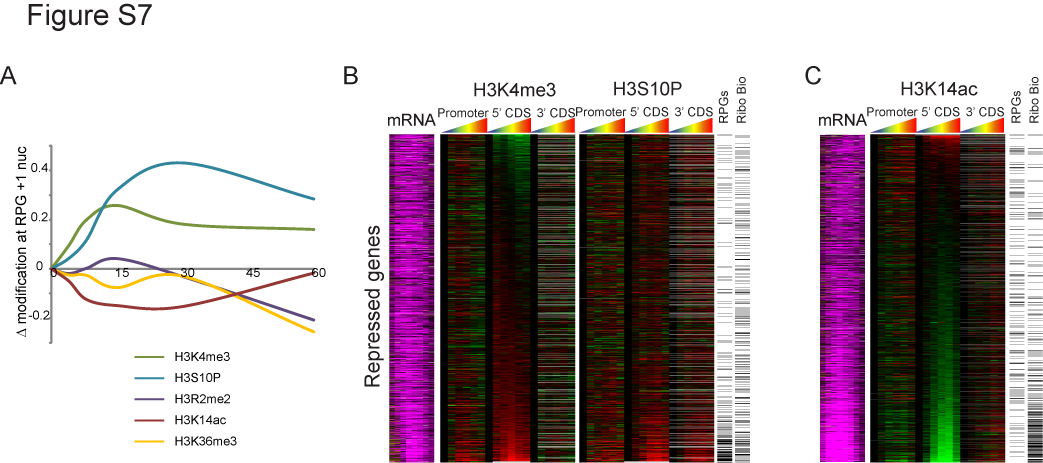

Supplement: Figure S7 — Specific chromatin changes associated with RPG and Ribi repression. (A) Specificity of H3K4me3 gain at RPG 5′ ends. Probes corresponding to the +1 nucleosome at RPGs were analyzed specifically, and time course data for all five modifications are shown as indicated. (B) The majority of diamide-repressed genes that gain H3K4me3 are RPGs. Diamide-repressed genes are sorted by the 5′ change in H3K4me3, with GO annotations shown in the right panel as indicated. (C) The majority of diamide-repressed genes that lose 5′ H3K14ac are Ribi genes. As in (B), but with genes sorted by 5′ change in H3K14ac. (TIF) [file pbio.1001369.s007.tif]
